# Encapsulation of Nanoparticles with Statistical Copolymers with Different Surface Charges and Analysis of Their Interactions with Proteins and Cells

**DOI:** 10.3390/ijms25105539

**Published:** 2024-05-19

**Authors:** Saad Megahed, Nicole Wutke, Yang Liu, Markus Klapper, Florian Schulz, Neus Feliu, Wolfgang J. Parak

**Affiliations:** 1 Fachbereich Physik, Universität Hamburg, Luruper Chaussee 149, 22761 Hamburg, Germany; smegahed@azhar.edu.eg (S.M.); yliu@physnet.uni-hamburg.de (Y.L.); florian.schulz@physik.uni-hamburg.de (F.S.); 2 Physics Department, Faculty of Science, Al-Azhar University, Cairo 11884, Egypt; 3 Max Planck Institute für Polymerforschung, 55128 Mainz, Germany; wutke@mpip-mainz.mpg.de (N.W.); klapper@mpip-mainz.mpg.de (M.K.); 4 Zentrum für Angewandte Nanotechnologie CAN, Fraunhofer-Institut für Angewandte Polymerforschung IAP, 20146 Hamburg, Germany; neus.feliu@physnet.uni-hamburg.de

**Keywords:** nanoparticles, amphiphilic polymers, protein corona, cellular uptake

## Abstract

Encapsulation with polymers is a well-known strategy to stabilize and functionalize nanomaterials and tune their physicochemical properties. Amphiphilic copolymers are promising in this context, but their structural diversity and complexity also make understanding and predicting their behavior challenging. This is particularly the case in complex media which are relevant for intended applications in medicine and nanobiotechnology. Here, we studied the encapsulation of gold nanoparticles and quantum dots with amphiphilic copolymers differing in their charge and molecular structure. Protein adsorption to the nanoconjugates was studied with fluorescence correlation spectroscopy, and their surface activity was studied with dynamic interfacial tensiometry. Encapsulation of the nanoparticles without affecting their characteristic properties was possible with all tested polymers and provided good stabilization. However, the interaction with proteins and cells significantly depended on structural details. We identified statistical copolymers providing strongly reduced protein adsorption and low unspecific cellular uptake. Interestingly, different zwitterionic amphiphilic copolymers showed substantial differences in their resulting bio-repulsive properties. Among the polymers tested herein, statistical copolymers with sulfobetaine and phosphatidylcholine sidechains performed better than copolymers with carboxylic acid- and dimethylamino-terminated sidechains.

## 1. Introduction

In nanomedicine and nanobiotechnology, polymers are commonly employed to stabilize and functionalize nanomaterials [1,2,3,4,5,6,7]. Their structure can be tuned to tailor their physicochemical properties, such as polarity and charge density. In contrast to small stabilizing ligands, they usually provide better steric stabilization, resulting in improved colloidal stability, in particular in aqueous solutions at high ionic strengths, over a wide pH range, and in complex environments [8,9,10,11,12,13]. The structural variety of polymers leads to various coating strategies for nanoparticles (NPs), including direct binding to the nanomaterials’ surfaces via suitable functional groups or encapsulation in layer-by-layer or micellar structures [2,14,15,16,17,18,19,20]. Here, many encapsulation strategies have the advantage that they do not depend on a suitable binding chemistry of the polymer ligand. Thus, a variety of loadings can be encapsulated, including different materials, sizes, and mixtures of nanomaterials.

Amphiphilic copolymers have been demonstrated as effective agents for the encapsulation of nanomaterials [16,21,22,23]. For instance, poly(isobutylene-*alt*-maleic anhydride) (PMA)-based polymers can encapsulate various nanomaterials, and the anhydride chemistry of the backbone allows for further functionalization or tuning of their properties. In nanobiotechnology, it is well established that protein adsorption, often referred to as protein corona formation, must be managed [24,25,26,27,28,29,30,31,32]. This is a complex process that delicately depends on the physicochemical properties of both the material and the binding properties, as well as the composition of the medium/matrix. Two prominent strategies to reduce unwanted protein adsorption are PEGylation (the binding of poly(ethylene glycol) (PEG)-based ligands to nanomaterials) [3,33,34,35,36,37] on the one hand and the use of small zwitterionic ligands on the other [38,39,40,41,42]. Even though these strategies seem to be very different, relying on uncharged polymers on the one hand or on small mixed-charged molecules on the other, they are both based on a strong hydration of the ligand shell for minimized protein binding [43,44]. There is an additional entropy-based reason for the protein-repelling nature of zwitterionic surfaces [39]. Zwitterionic surfaces are neutral and thus there is no layer of adsorbed counter ions. In this way, in contrast to electrostatically mediated protein adsorption, where binding is driven by the pairing of charges of opposite polarity and causes counter ions to be released from both binding partners (the surface and the proteins), this effect does not take place in the case of zwitterionic surfaces, i.e., adsorption of proteins does not lead to an increase in entropy based on the release of counter ions [39,45,46]. Recently, zwitterionic amphiphilic copolymers have been presented; these combine the advantages of versatile polymer encapsulation chemistry and minimized protein adsorption [47,48]. Very recently, it has also been shown that the detailed surface charge distributions of PEGylated nanoparticles (with mixed PEGs with terminal residues of different polarity) strongly affect their affinity to different proteins [49]. We were therefore interested in the effects of different copolymer surface chemistries for the coating of NPs on the NPs’ colloidal stability, protein adsorption properties, and uptake by cells. We prepared a range of amphiphilic copolymers with different properties (negatively charged, positively charged, or zwitterionic) and encapsulated different nanoparticles with these polymers, namely gold nanoparticles (Au NPs) and quantum dots (QDs). The properties of the resulting NPs were studied with dynamic light scattering (DLS), zeta-potential measurements, and dynamic interfacial tensiometry (DIT). Adsorption of proteins to the NPs was quantified with fluorescence correlation spectroscopy (FCS), and their interaction with cells was determined using viability tests and uptake studies. While our previous studies were focused on colloidal stability, we herein explore the protein adsorption, the physicochemical parameters that are associated with it, and the consequences for cellular uptake. Despite the many involved parameters (which challenge a systematic approach), we were able to extract some clear tendencies. Most importantly, zwitterionic polymers have proven to be favorable for minimized protein adsorption and very low unspecific uptake, but the details of the polymer structure are important. Statistical copolymers with sulfobetaine and phosphatidylcholine sidechains yielded better results than copolymers with carboxylic acid- and dimethylamino-terminated sidechains.

## 2. Results and Discussion

### 2.1. Particle Synthesis and Encapsulation

**Figure 1:**
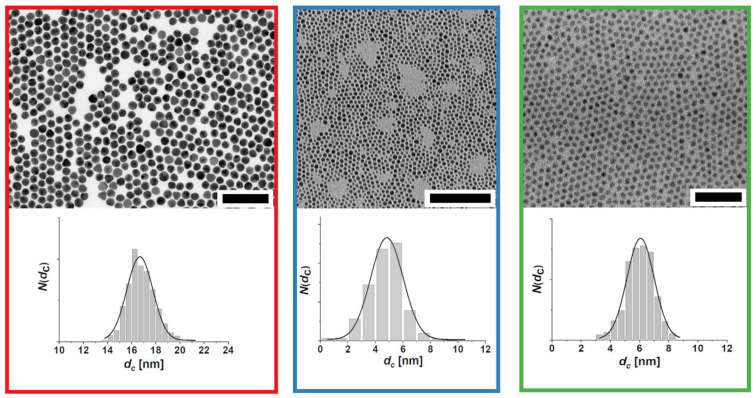
Exemplary TEM measurements of the nanoparticles used in this study and the respective distributions *N*(d_c_) of the core diameters *d_c_*. Red: Au NPs *d_c_*~17 nm (scale bar: 100 nm); blue: Au NPs *d_c_*~5 nm (scale bar: 100 nm); green: QDs *d_c_*~6 nm (scale bar: 50 nm).

Au NP with mean core diameters of *d_c_*~5 nm and *d_c_*~17 nm were synthesized using established protocols [18,50,51]. A detailed description of all procedures (including the basic characterization of all nanoparticles used in this study) is provided in the Supporting Information (Figures S1–S6). Quantum dots (QD; CdSe/CdS/ZnS core/shell/shell) with a mean diameter of *d_c_*~6 nm were provided by Fraunhofer IAP-CAN. Exemplary transmission electron microscopy (TEM) measurements of the inorganic nanoparticle cores are shown in Figure 1.

**Figure 2:**
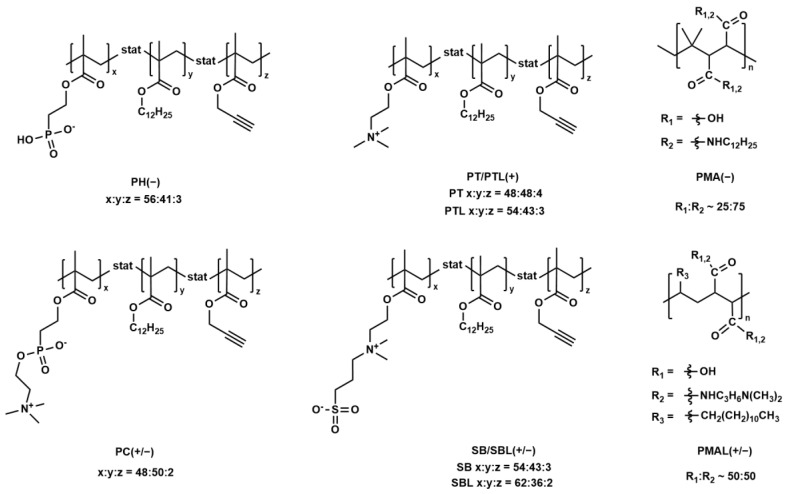
Structure of the amphiphilic copolymers. PMA(−) and PMAL(+/−) are alternating copolymers. PT(+/−)/PTL(+/−) and SB(+/−)/SBL(+/−) differ in molecular weight.

**Figure 3:**
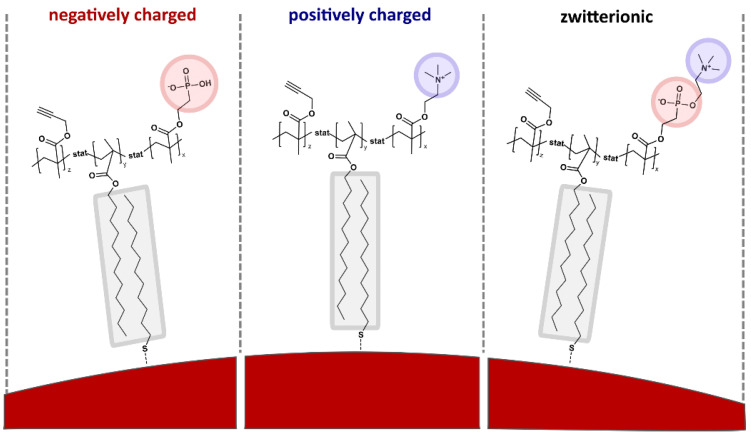
A range of different amphiphilic copolymers was compared as illustrated for three examples and the case of gold nanoparticles (Au NP). The hydrophobic lauryl (C_12_) side chains form an inner hydrophobic layer with the according alkyl chains of the dodecanethiol ligands bound to the gold surface. This hydrophobic layer, highlighted in grey, is stabilized by intermolecular attractive van der Waals interactions. Phosphonic acid- or trimethylammonium-terminated, or zwitterionic (here phosphatidylcholine-based) side chains provide a negatively charged (highlighted in red), positively charged (highlighted in blue), or zwitterionic surface.

The encapsulation procedure was based on established protocols [18,23]. All polymers discussed herein feature hydrophobic lauryl side chains facilitating the strong physisorption of the copolymers to the nanoparticles with hydrophobic coatings in chloroform. The structure of all copolymers is shown in Figure 2 and the general surface structure is sketched in Figure 3. Additional information is summarized in Table 1.

We compared statistical copolymers with a negative (PH(−)) or positive (PT(+)) charge (via phosphate or ammonium residues) or zwitterionic side chains. Among the latter, we compared sulfobetaine (SB(+/−)) and phosphatidylcholine (PC(+/−))-based side chains. Additionally, we tested PMA(−), which is a standard amphiphilic copolymer coating (as mentioned above) and PMAL(+/−), which is an amphiphilic zwitterionic copolymer used for the solubilization of integral membrane proteins [52]. The different formal charges in PMAL(+/−) are on different side chains, whereas in SB(+/−) and PC(+/−) they are located in the same side chain. These structural features might play a role in the different behaviors of these polymers, which will be discussed throughout the manuscript.

### 2.2. Colloidal Stability

**Figure 4:**
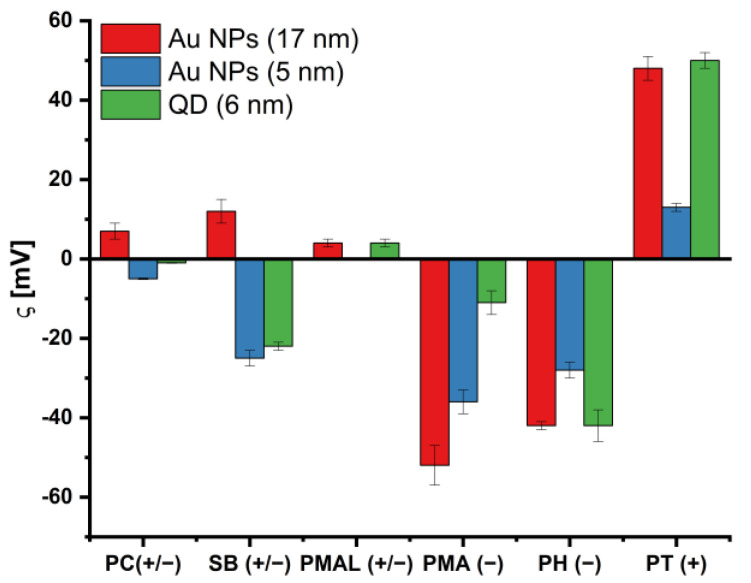
*ζ*-potentials of all conjugates as recorded in Milli-Q water.

The stability of the nanoparticles with the different coatings was tested with UV/vis absorption spectroscopy and with dynamic light scattering (DLS) (Supporting Information Figures S7–S9). Additionally, *ζ*-potentials were also measured (Figure 4), which provide an idea of the effective charge of the NPs in solution. Notably, the *ζ*-potentials do not necessarily reflect the surface charge/potential of the conjugates but refer to the potential at the shearing plane when the particles are moved in an external electric field. In particular, for polymer coatings this means that measured *ζ*-potentials are not necessarily the ones we would expect based on the formal charge, as discussed recently [33].

In the case of plasmonic gold nanoparticles (Au NPs), aggregation leads to plasmonic coupling, which drastically changes the optical properties [53]. Likewise, dissolution of the particles or strong changes in their dielectric environment affect their optical properties and therefore can be monitored with UV/vis absorption spectroscopy. Aggregation also leads to increased hydrodynamic diameters, as measured using DLS.

For the Au NP samples studied herein, we found no indication of destabilization, neither with UV/vis absorption spectroscopy nor with DLS (Supporting Information, Figures S1–S4), confirming that the coating approach with amphiphilic polymers featuring grafted hydrophobic sidechains can be applied in general to a variety of different polymers. In the presence of high NaCl concentrations (>1 M), some samples showed slight indications of some aggregation (e.g., PT(+)-coated Au NPs with *d_c_*~17 nm diameter and PH(−)-coated Au NPs with *d_c_*~5 nm; see Supporting Information, Figures S7–S9), but the majority of samples were stable.

Because electrostatic interactions are screened at high ionic strengths, this observation indicates that the polymer coatings provide both steric and—in some cases—additional electrostatic colloidal stabilization. This is crucial for applications in nanobiotechnology, which commonly require media with high ionic strengths. Similar observations were made for the polymer-coated QDs. For those, absorption and emission spectra were recorded, and in addition to the DLS measurements, fluorescence correlation spectroscopy (FCS) was used to determine the hydrodynamic diameter *d_h_*. Again, the optical properties showed no indication of aggregation or destabilization (Supporting Information, Figure S5) and the FCS and DLS measurements were in good agreement with no indications of aggregation as well (Supporting Information, Figure S6 and Table S3).

The increase in size due to the polymer coatings (e.g., the difference between the hydrodynamic radius *r_h_ = d_h_*/2 and the core radius *r_c_ = d_c_*/2 was in the range 2–9 nm for all tested conjugates, a reasonable range for coatings with polymers in this range of molecular weights (4000–22,000 g/mol) [54]. However, there was no clear correlation between polymer molecular weight and resulting ligand shell thickness. This is to be expected, because with the coating strategy employed herein, the polymers are “wrapped” around the particles and not, e.g., in a brush conformation, as could be the case for polymers grafted to the NPs surface directly [7].

The *ζ*-potentials of the functionalized nanoparticles in water were measured. We note that no *ζ*-potentials of the plain polymers (i.e., with no wrapping around the NPs) were measured. We also note that the *ζ*-potentials in cell medium can differ from those in water due to the different buffer conditions (ionic strength, pH, and presence of various molecules).

The *ζ*-potentials (as summarized in Figure 4) are in good agreement with the expected surface charge based on the character of the polymers (cationic, anionic, or zwitterionic), except for the sulfobetaine-derivatives (SB(+/−) and SBL(+/−) (the measured values are also provided as tables in the Supporting Information, Tables S1–S3). An anionic character (despite being formally uncharged) has been shown for zwitterionic micelles; it was shown to be more pronounced for sulfobetaines than for phosphatidylcholines [55]. This has also been found for zwitterionic block–copolymer coatings, where a negative zeta potential was observed over the pH range 1–10 for sulfobetaine derivatives [56]. This observation was explained by the strongly acidic character of sulfonic acids, which have reported pK_a_ values well below <1, often even in the negative range [57]. For zwitterionic amphiphilic polymers with phosphatidylcholine sidechains, the *ζ*-potentials were around 0 mV, in accordance with the formal net charge. We also note that the values as reported here are not fully quantitative. In the coatings presented herein, there are several possibilities for batch-to-batch variations. First, there may be batch-to-batch diameter and uniformity variations in the synthesis of the core NPs, then there may be batch-to-batch variations in the polymer synthesis, in the polymer coating procedure, and in the purification steps. The obtained data thus allow for qualitative conclusions, but they do not allow for conclusions based on, for example, differences due to the core size of the Au NPs (e.g., Au NPs (17 nm) versus Au NPs (5 nm)). To relate physicochemical characterization data to parameters such as the core size, a much larger set of NPs with different core sizes and core materials would have been required.

### 2.3. Interfacial Tension

**Figure 5:**
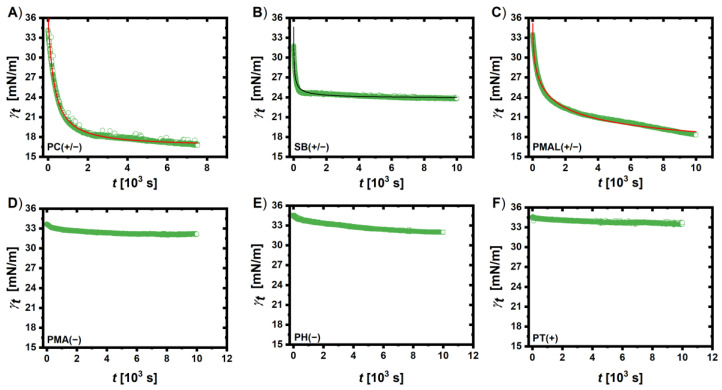
Interfacial tension (water–toluene) as function of time, γ_t_, modulated by polymer-coated QDs dispersed in the water phase. QDs were coated with (**A**) PC(+/−), (**B**) SB(+/−), (**C**) PMAL(+/−), (**D**) PMA(−), (**E**) PH(−), and (**F**) PT(+). The red lines are fits with the Hua and Rosen equation where applicable [62].

Dynamic interfacial tension (DIT) measurements using the pendant drop method (water in toluene) were conducted to determine the surface activity of the nanoconjugates (details of the measurement and fitting procedure are provided in the Supporting Information, Supporting Note S8) following the strategy of a previous publication [58]. The adsorption of the conjugates at the liquid–liquid interface decreases the interfacial tension, and the time taken to reach an equilibrium value depends on the diffusion and adsorption rates. Rana et al. found a linear correlation of hydrophobicity and surface tension at meso-equilibrium for small gold NPs [59]. With increasing hydrophobicity of the coating ligands, the interfacial tension at meso-equilibrium decreased and this equilibrium was reached faster, as expressed by the maximum surface tension decay rate *ν*_max_. Likewise, the hydrophobicity of the NPs affected the binding stoichiometry with proteins and thus is relevant for the interactions with proteins in general. Generally, an increased adsorption of proteins is observed with increasing hydrophobicity of the surface [60,61]. The results of the DIT measurements are presented in Figure 5 for the polymer-coated QDs and in the Supporting Information, Figure S10 for Au NPs (17 nm). A summary of the fitting results for all measurements is also provided as Supporting Information (Tables S4 and S5).

In the case of QDs, all tested polymer coatings with a formal charge (PMA(−), PH(−), and PT(+)) failed to change the interfacial tension *γ* substantially. Starting from a value of 34–35 mNm^−1^, the final values at the end of the measurements *γ*_eq_ were >32 mNm^−1^. In contrast, the zwitterionic polymers decreased the interfacial tension substantially, down to 16 mNm^−1^. Interestingly, the zwitterionic polymer coatings with a *ζ*-potential close to zero, PMAL(+/−) and PC(+/−), decreased the interfacial tension to this value, whereas the sulfobetaine-based coating (SB(+/−)), with an anionic character in the *ζ*-potential measurements, decreased the interfacial tension significantly less (*γ* _eq_ = 24.3 mNm^−1^) but much faster: *ν*_max_ = 85 × 10^−3^ mN(ms)^−1^ for SB(+/−), 18 × 10^−3^ mN(ms)^−1^ for PC(+/−), and 5 × 10^−3^ mN(ms)^−1^ for PMAL(+/−). This indicates a fast diffusion and adsorption of the SB(+/−) conjugates at the interfacial layer, but a less stable structure of the interfacial film, possibly due to electrostatic repulsion. The (formal) charge neutrality of the zwitterionic coatings allows for their assembly at the toluene–water interface, thus modulating the interfacial tension similar to surfactants. However, zwitterionic moieties are not hydrophobic but highly hydrophilic, so it would be misleading to conclude here that the zwitterionic coatings provide the core particles with a hydrophobic character as in the study of Rana et al. In addition, the qualitative nature of the results due to batch-to-batch variations needs to be stressed again. As will be discussed in the following for the Au NPs, some of the coatings lead to different behaviors, and our data thus do not allow for quantitative conclusions.

For the larger Au NPs (*d* = 17 nm), the results partly differed (cf., Supporting Information, Figure S10 and Table S5). Here, it was not only the tested zwitterionic coatings (PC(+/−) and SBL(+/−) that decreased the interfacial tension: the anionic coatings PMA(−) and PH(−) did as well. This might be due to curvature effects resulting from a different coating structure, which in turn may not have depended on the different nature of the 6 different types of used polymer but may also be influenced by batch-to-batch variations. The cationic coating did not decrease the interfacial tension, similar to the behavior of the respective QDs. Comparing the PC(+/−) coating for QDs and Au NPs (*d* = 17 nm), the time to reach meso-equilibrium was longer for the Au NPs (*d* = 17 nm), which might be explained with the larger hydrodynamic diameter and therefore slower diffusion.

Taken together, the DIT results confirm the higher surface activity of the zwitterionic coatings. This can reasonably be expected to affect protein adsorption on the NPs. To confirm this, we used fluorescence correlation spectroscopy (FCS) to measure the protein adsorption to the QD samples directly via the increase in their hydrodynamic radii over time.

### 2.4. Fluorescence Correlation Spectroscopy

**Figure 6:**
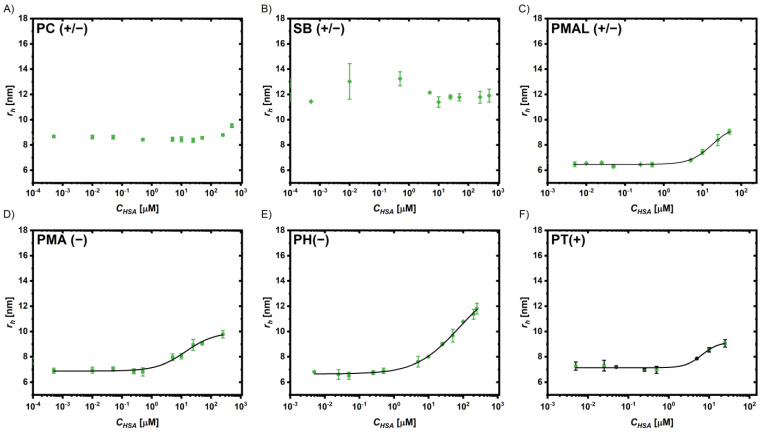
Change of the hydrodynamic radii *r*_h_ of the QDs coated with (**A**) PC(+/−), (**B**) SB(+/−), (**C**) PMAL (+/−), (**D**) PMA (−), (**E**) PH(−), and (**F**) PT(+) in the presence of different HSA concentrations *c*_HSA_. The black line represents a fit based on the Hill model.

**Figure 7:**
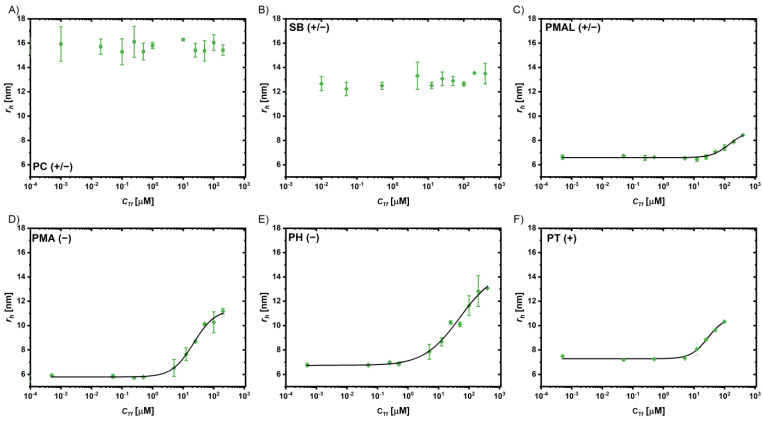
Change of the hydrodynamic radii *r*_h_ of the QDs coated with (**A**) PC(+/−), (**B**) SB(+/−), (**C**) PMAL (+/−), (**D**) PMA (−), (**E**) PH(−), and (**F**) PT(+) in the presence of different Tf concentrations *c*_Tf_. The black line represents a fit based on the Hill model. Note that the *r_h_* data at low protein concentration are not the same as the ones in Figure 6, which is due to batch-to-batch variations.

FCS has been demonstrated as an excellent tool to study protein adsorption to fluorescent NPs like QDs [63,64]. It allows us to measure the characteristic diffusion time *τ*_D_, which can be used to calculate the diffusion constant *D* and, via the Stokes–Einstein relation, the hydrodynamic radii *r*_h_ = *d_h_/2*. Considering the volume of the proteins, the number of bound proteins can be estimated based on geometry considerations, as described previously [28,63]. By measuring the dependence of the protein concentration *c_P_* on the hydrodynamic radius *r_h_* of the NPs (which upon adsorption of proteins increases), the Hill equation can be used to fit the data and obtain the apparent dissociation constant *K*_D_ and the maximum number of adsorbed proteins *N*_max_ as fit parameters [28,65]. A detailed description of the experiments and analysis is provided in the Supporting Information (Supporting Note S9). The adsorption of the abundant serum proteins, human serum albumin (HSA) and transferrin (Tf), were tested. The data and fits are shown in Figure 6 for HSA and in Figure 7 for Tf absorption; the fitting parameters are provided in the Supporting Information, Tables S6 and S7. Note that the Au NPs could not be assessed via FCS due to lack of fluorescence.

For the coatings PMA(−) and PT(+), a comparably strong binding in a similar range was observed for both, with the *K*_D_ values in the lower micromolar range (*K*_D_(PMA(−)-HSA) = 21 µM, *K*_D_(PT(+)-HSA) = 7.5 µM, and *K*_D_(PMA(−)-Tf) and *K*_D_(PT(+)-Tf) both = 35 µM). For the PH(−) coatings, the binding was weaker (*K*_D_ > 100 µM) and the error for the fit parameters was very high. At the same time, the number of adsorbed proteins *N*_max_ was highest for these coatings (106 for HSA and 38 for Tf); again, these values have to be interpreted with care due to significant uncertainty in the fit, as the *K_D_* value is close to the highest experimentally accessed protein concentration *c_p_*. Considering the geometry of the proteins and the coated QDs, a monolayer could not contain much more than ~20 HSA molecules and no more than ~10 of the larger Tf molecules (see the Supporting Information, Supporting Note S9 and Figure S11). The other tested anionic coating PMA(−) also adsorbed a higher number of proteins than expected for a monolayer, but the numbers were much closer (*N*_max_(HSA) = 40, *N*_max_(Tf) = 17). The cationic coating (PT)+) adsorbed the proteins with high affinity (in particular HSA) but in low numbers in the range of monolayer coverage (*N*_max_(HSA) = 17, *N*_max_(Tf) = 11). The isoelectric point of HSA is lower (4.7–5.4) than that of transferrin (5.4–6.2), i.e., at a given pH, HSA is more negatively charged, which can also be observed from the corresponding surface charge distributions (Supporting Information, Figure S11). The strong binding of HSA to the cationic coating might thus be explained by attractive electrostatic interactions. It has to be stressed, however, that even though electrostatic interactions are known to affect protein adsorption, additional effects like hydrophobicity and non-covalent chemical interactions have to be taken into account when interpreting protein–nanoparticle interactions [28,31,66,67]. The results are in line with previous data showing high levels of protein adsorption to negatively and positively charged NPs [68].

In contrast, for the zwitterionic coatings based on the statistic amphiphilic copolymers SB(+/−) and PC(+/−), no significant protein adsorption was observed in the FCS experiments. This confirms that these zwitterionic coatings indeed strongly reduce protein adsorption. The effective suppression of protein adsorption by the zwitterionic polymer coatings PC(+/−) and SB(+/−) is in agreement with previous reports [69]. On the other hand, the coating based on the zwitterionic polymer PMAL(+/−) was less effective in reducing the adsorption of HSA and Tf. In SB(+/−) and PC(+/−), the ionic moieties are close together, separated by only two methylene units; however, in PMAL(+/−), they are in different sidechains of the polymer backbone. In addition, the ionic species contributing the negative charge are different, phosphate and sulfonate in PC(+/−) and SB(+/−) vs. carboxylic acids in PMAL(+/−).

Since protein adsorption strongly affects the interactions of NPs with cells, as a next step we tested for these interactions in a set of experiments.

### 2.5. Cell Viability and Uptake

First, cell viability in the presence of the different NPs and coatings was evaluated with the resazurin assay (Supporting Information, Figure S12), similar to previous reports [40,70,71]. Cytotoxicity can be due to the release of cytotoxic components of the core (e.g., Cd^2+^ in the case of QDs, including core-shell QDs [72,73]) that can be reduced by a suitable coating [74]. On the other hand, coating ligands can also contribute to the cytotoxicity of NPs, a prominent example being the cetyltrimethylammonium bromide or chloride ligands on some gold nanomaterials [75,76]. Cell viability assays are an essential complement to NP uptake studies by cells, as there is a mutual influence. In case of high cellular uptake of NPs, cytotoxic effects may be higher. In turn, in case of high toxicity, cells are severely impaired up to cellular death, which in turn would reduce further uptake of NPs.

In the case of the Au NPs within the investigated range of NP concentrations c_NP_, no reduction in the viability of HeLa cells was found except for the case of Au NPs (5 nm) coated with PT(+) at the highest used concentration. In the case of QDs, the highest used NP concentrations reached a level where viability was reduced. This was again the case for the PT(+) coating and also for PMAL(+/−). This is in line with suggestions that positively charged NPs can have a stronger effect on cellular viability than other NPs with different coatings [77,78], although there are also contradictory reports in the literature [79].

The cellular uptake of the different NPs and coatings was determined with inductively coupled plasma mass spectrometry (ICP-MS)-based elemental analysis. Note that we assumed that NPs found in cells were endocytosed NPs, and that the amount of NPs adherent only to the outer cell membrane plays no significant role after washing of the cells before ICP-MS analysis.

**Figure 8:**
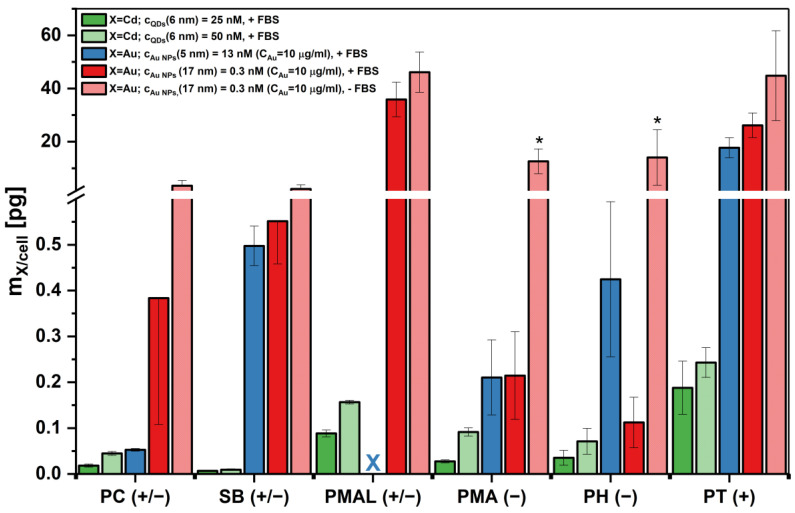
Cellular uptake of the NPs with different coatings by HeLa cells after 24 h exposure as determined by elemental analysis in terms of mass *m*_X_ of element X found per cell (X = Cd for QDs and X = Au for Au NPs). The QDs are compared at different exposure concentrations (dark green: *c*_NP_ = 25 nM; light green: 50 nM; both in the presence of FBS: +FBS). For the larger Au NPs (17 nm) the uptake is compared at the same concentration in the presence (dark red, +FBS) or absence (light red, −FBS) of FBS supplement. Meanwhile, for the Au NPs, the molar concentrations c_NP_ are different for the 5 nm and 17 nm NPs (13 vs. 0.3 nM), and the Au exposure concentration in terms of Au is the same in both cases: C_Au_ = 10 μg/mL. Data from at least three independent measurements are shown with the standard deviations of the mean. Significance tested with Student’s *t*-test (* *p* < 0.05). For the “x”, no data could be measured.

For the QDs, a number of control experiments was performed with flow cytometry utilizing their fluorescence emissions. All procedures are described in detail in the Supporting Information (Supporting Note S10). Figure 8 summarizes the uptake after 24 h for different conjugates (core and coating) and varying conditions such as concentration and the presence or absence of fetal bovine serum (FBS) supplement (i.e., serum proteins). In line with previous findings, NP uptake without the presence of FBS was found to be higher than in FBS-supplemented conditions (see also the Supporting Information, Figures S13 and S14 for the complementary experiments) [80,81].

In the case of QDs, the lowest uptake was observed for the zwitterionic coatings SB(+/−) and PC(+/−), followed by a significantly higher uptake of the anionic coatings PMA(−) and PH(−). The highest uptake was observed for PT(+). Despite the zwitterionic coating, there was also significant uptake of PMAL(+/−)-coated QDs. For the Au NPs, a similar trend was observed.

This shows clearly that the effect of zwitterionic coating cannot be generalized and depends on the chemical structure of the coating. In both the protein adsorption and the cellular uptake experiments, PMAL(+/−)-coated NPs showed behavior more similar to the charged NPs than to the other zwitterionic NPs coated with SB(+/−) and PC(+/−).

One hypothesis can be based on the ruling out of counter ion displacement upon protein adsorption, as mentioned above [39]. If positive and negative charges sit on the same ligand at close distance (i.e., PC(+/−) and SB(+/−)), there will be no adsorbed counter ions, as both charges in close proximity would share the same counter ions. This results in no counter ions due to the cancelling of the net charge that occurs when the negative and the positive charge are close to each other at the same ligand. In contrast, for mixed charged ligands, the distance between negative and positive charges may be bigger, thus there would be local counter ion clouds around these charges, which upon protein binding would be displaced, increasing entropy [39]. We thus speculate that protein repulsion works better for zwitterionic polymers where positive and negative charge pairs share the same ligand chain (e.g., PC(+/−) and SB(+/−)), in contrast to zwitterionic polymers where the positive and negative charge pairs sit on separate ligand chains (e.g., PMAL(+/−)). In order to test this hypothesis, an additional surface coating based on edelfosine (EDLF (+/−), 2-methoxy-3-(octadecyloxy)propyl-2-(trimethylazaniumyl)ethyl phosphate) (cf. Figure S15A) was used. In contrast to the other amphiphilic polymers used in this study, EDLF (+/−) is a single amphiphilic ligand. Similar to the amphiphilic polymers shown in Figure 2, the hydrophobic tail may intercalate the hydrophobic ligand layer around NPs, causing the hydrophilic head group to point towards the solution and rendering the hydrophobically capped NPs hydrophilic and thus water-soluble. The hydrodynamic diameters shown in Table S8 show that the coating yielded NPs well dispersed in the aqueous phase. The protein adsorption data shown in Figure S15B demonstrate low protein adsorption of HSA and Tf, more similar to the case of PC(+/−) and SB(+/−) coatings than to PMAL(+/−) coating (cf. Figure S15B, Figure 6 and Figure 7). Meanwhile, the EDLF (+/−)-coated NPs show some toxicity (cf. Figure S15C), and their uptake by cells is reduced (cf. Figure S15D vs. Figure 8). Again, the behavior of the EDLF (+/−) coating on the cellular uptake of NPs is more similar to PC(+/−) and SB(+/−) than to the PMAL(+/−) coating. While the hypothesis of the influence of the difference in positive and negative charges on the same versus on different ligand chains is not fully proven with these data, results are supportive of this hypothesis.

Taken together, all coatings tested herein provide a good stabilization for different nanoparticles: small QDs and Au NPs (5 nm and 17 nm). DIT measurements revealed that the surface activity of the zwitterionic coatings is higher in general. Even though they are very hydrophilic, their net formal charge is zero (which does not imply that their zeta potential would be zero in general), allowing for their assembly at the water–toluene interface, thereby modulating the interfacial tension. One might speculate that some of the hydrophobic lauryl sidechains in the copolymers orient towards the toluene phase, thereby stabilizing the interfacial assembly similar to zwitterionic tensides. Via FCS experiments we found that the protein adsorption differs for the different charged coatings, even for those with the same formal charge, underlining that protein adsorption cannot be understood based on electrostatic interactions alone.

Remarkably, the zwitterionic coating PMAL(+/−) did not prevent protein adsorption to the same extent as was found for the statistical copolymers SB(+/−) and PC(+/−), where no significant adsorption was observed. A similar observation was made in the NP uptake by cells experiments, where reduced uptake was observed only for the PC(+/−) and SB(+/−)-coated NPs.

## 3. Materials and Methods

The syntheses used in this study are based on previous work and therefore only briefly summarized here. For a detailed description of all syntheses and procedures we refer to the Supporting Information.

*Nanoparticle syntheses.* Au NPs with a mean core diameter of *d_c_*~17 nm were synthesized based on the protocol described by Bastús et al. [51]. Au NPs with a mean core diameter of *d_c_*~5 nm were synthesized with the Brust-synthesis [50]. The QDs (core/shell/shell) CdSe/CdS/ZnS QD Lot No. SAB-0-365-6 were provided by the Fraunhofer Center for Applied Nanotechnology CAN (Hamburg, Germany).

*Polymer syntheses.* All polymer syntheses and modifications were performed according to previously published protocols. The PMA derivatives were synthesized based on the concept by Pellegrino et al. [23] and as described in Lin et al. [21] and Hühn et al. [18]. The statistical copolymers were synthesized as described in Geidel et al. [82], Hühn et al. [68], and Valdeperez et al. [47].

*Encapsulation of nanoparticles.* The encapsulation of the different NPs was performed using the general protocol described in Hühn et al. [18].

*Instrumentation.* DLS and ζ-potential measurements were performed with a Zetasizer Nano ZS system (Malvern Panalytical, Worcestershire, UK), which employs a He–Ne laser (4.0 mW, 633 nm). TEM measurements were performed with a JEOL JEM-1011 at 100 keV (JEOL, Tokyo, Japan); UV/Vis absorption spectroscopy was performed using a Cary 60 spectrophotometer (Agilent Technologies, Santa Clara, CA, USA). Dynamic interfacial tension measurements were recorded with a Drop Shape Analysis system (DSA30S, Krüss, Hamburg, Germany). Protein adsorption to the encapsulated QDs was measured with FCS using confocal light scanning microscopy (CLSM) (LSM 880, Zeiss, Oberkochen, Germany) with a Zeiss Plan-Apochromat 40×/1.0 water (WD 2.5 mm) objective via the approach described by Liedl et al. [64] and Röcker et al. [63]. Elemental mass concentrations from cell uptake experiments were measured with an Agilent 7700 ICP-MS setup (Agilent Technologies, Santa Clara, CA, USA). The calibration curves were prepared with corresponding elemental standards (Carl Roth, Karlsruhe, Germany). Complementary flow cytometry was performed using a BD LSRFortessa^TM^ (BD Bioscience, Franklin Lakes, NJ, USA).

## 4. Conclusions

We compared amphiphilic copolymers with different formal net charges for the coating of nanoparticles. Based on DIT, FCS, and cellular uptake experiments, we identified zwitterionic coatings as candidates to achieve minimum protein adsorption and minimum nonspecific cellular uptake. Zwitterionic coatings with sulfobetaine- (SB(+/−) and phosphatidylcholine- (PC(+/−) sidechains provided the best results in this respect. Coating with another zwitterionic polymer, i.e., PMAL(+/−), did not show the same effect. This finding also points at a possible difference between statistical and alternating copolymers that could be studied in more detail in future studies. Amphiphilic copolymers of different classes (statistical vs. block copolymers) have previously been observed to perform differently in nanoparticles synthesis and encapsulation [83]. The detailed structure of copolymers can affect the dynamics of micelle formation and consequently the resulting structures, for instance the yield of well-defined micelles. Similarly, the encapsulation of nanoparticles might be affected by copolymer structure. Thus, the conformations of the adsorbed polymers can be affected. However, in the present study the different copolymer types also differed in the presentation and type of functional groups that establish the zwitterionic character; therefore, we cannot draw any conclusions regarding these effects.

The coating strategy utilizing amphiphilic copolymers is general in that different NPs can be encapsulated and versatile because via polymer synthesis, properties like the ratio of the sidechains and the molecular weights of the coating ligands can be varied and optimized for the desired material. It is important to consider the results of this study as qualitative, as for improved quantitative analysis, a larger library of NPs and a more detailed analysis of batch-to-batch variations would be required.

## Figures and Tables

**Table 1:** Polymers used in the present study.

Full Name of Polymer	Abbreviation	*M*_w_ [g/mol]	x:y:z ^a^	Charge
PTMAEMA-*stat*-PLMA-*stat*-PPgMA ^b^	PT	15,500	48:48:04	+
	PTL	7200	54:43:03	+
PSB-*stat*-PLMA-*stat*-PPgMA ^b^	SBL	3900	62:36:02	+/−
	SB	7200	54:43:03	+/−
PMPC-*stat*-PLMA-*stat*-PPgMA ^b^	PC	22,000	48:50:02	+/−
PMAPHOS-*stat*-PLMA-*stat*-PPgMA ^b^	PH	11,000	56:41:03	−
poly(isobutylene-*alt*-maleic anhydride)-*graft*-dodecylamine	PMA	11,400	25:75	−
poly(maleic anhydride-*alt*-1-tetradecene)-3-(dimethylamino)-1-propylamine derivative	PMAL	12,000	50:50	+/−

^a^ x:y:z is the ratio of the hydrophilic (charged) to hydrophobic (PLMA) to functionalized (PPgMA) monomer units, respectively. ^b^ PTMAEMA: poly(2-(*N*,N,N-trimethylammonium)ethyl methacrylate). PLMA: poly(lauryl methacrylate). PPgMA: poly(propargyl methacrylate). PSB: poly (2-(*N*-3-sulfopropyl-*N*,*N*-dimethylammonium)ethyl methacrylate). PMPC: poly(2-(methacryloyloxy)ethylphosphorylcholine). PMAPHOS: poly(2-(methacryloyloxy)ethylphosphonic acid).

## Data Availability

Data are contained within the article and Supplementary Materials.

## Supplementary Materials

The following supporting information can be downloaded at: https://www.mdpi.com/article/10.3390/ijms25105539/s1. References [84,85,86,87,88,89,90,91,92,93,94,95,96] are cited in the supplementary materials.
