# Supplementary material for: Encapsulation of Nanoparticles with Statistical Copolymers with Different Surface Charges and Analysis of Their Interactions with Proteins and Cells
Source: Int J Mol Sci. 2024 May 19;25(10):5539. doi: 10.3390/ijms25105539 (PMC11122285; doi:10.3390/ijms25105539)
Supplement: Supplementary file 1 [file ijms-25-05539-s001.zip › ijms-2982896-supplementary.pdf]

SUPPORTING INFORMATION
for

Encapsulation of Nanoparticles with Statistical Copolymers with Different Surface Charges and Analysis of Their Interactions with Proteins and Cells

Saad Megahed [1,2], Nicole Wutke [3], Yang Liu [1], Markus Klapper [3], Florian Schulz [1], Neus Feliu [4] and Wolfgang J. Parak [1,*]

[1] Fachbereich Physik, Universität Hamburg, Luruper Chaussee 149, 22761 Hamburg, Germany
[2] Physics Department, Faculty of Science, Al-Azhar University, Cairo 11884, Egypt
[3] Max Planck Institute für Polymerforschung, 55128 Mainz, Germany
[4] Zentrum für Angewandte Nanotechnologie CAN, Fraunhofer-Institut für Angewandte Polymerforschung IAP, 20146 Hamburg, Germany
* Correspondence: wolfgang.parak@uni-hamburg.de

Content

S1. Materials

S2. Synthesis of hydrophobically capped gold nanoparticles (Au NPs)
S2.1. Synthesis of Au NPs (17 nm)
S2.2. Synthesis of Au NPs (5 nm)

S3. Amphiphilic polymers used in the present study

S4. Polymer coating of the nanoparticles

S5. Characterization of the polymer-coated gold nanoparticles
S5.1. UV-vis absorption spectroscopy
S5.2. Dynamic Light Scattering (DLS)

S6. Characterization of polymer-coated QDs
S6.1. UV-vis absorption and fluorescence spectroscopy
S6.2. Dynamic Light Scattering (DLS)

S7. Colloidal stability of the NPs in NaCl solutions

S8. Dynamical interfacial tension (IFT) measurements

S9. Fluorescence correlation spectroscopy (FCS) measurements

S10. Cell viability assays

S11. NP uptake by cells
S11.1. Inductively coupled plasma mass spectrometry (ICP-MS) quantification
S11.2. Flow cytometry quantification

S12. Control experiments with edelfosine

S13. References

S1. Materials.

Tetrachloroauric acid ($HAuCl_4$), trisodium citrate dihydrate, dodecylamine, tetraoctylammonium bromide (TOAB), NaOH, dodecanethiol (DDT), poly(isobutylene-*alt*-maleic anhydride), poly (maleic anhydride-*alt*-1-octadecene)-3-(dimethylamino)-1-propylamine derivative (PMAL), resazurin sodium salt, rhodamine 6G dye (Rh6G) were from Merck/Sigma Aldrich (Darmstadt, Germany), tetrahydrofuran (THF), chloroform, and hydrochloric acid (HCl) were purchased from Carl Roth (Karlsruhe, Germany). α-Methoxy-ω-mercapto-poly(ethylene glycol) (mPEG-SH) was from Rapp polymers (Tübingen, Germany). Dulbecco's modified eagle medium (DMEM), and trypsin-EDTA were from Thermo Fisher (Waltham, MA, USA). Concentrated nitric acid (HNO_3, 67%) and hydrochloric acid (HCl, 37%) were from Fisher Chemicals. The QDs (core/shell/shell) CdSe/CdS/ZnS QD Lot No. SAB-0-365-6 were provided by the Fraunhofer Center for Applied Nanotechnology CAN (Hamburg, Germany). Ultrapure water (MilliQ, 18.6 $M\Omega cm^{-1}$) was used for all procedures.

S2. Synthesis of hydrophobically capped gold nanoparticles (Au NPs).

S2.1. Synthesis of Au NPs (17 nm).

Spherical gold nanoparticles (NPs) were synthesized with established protocols.[18, 51, 84-85] Briefly, aqueous sodium citrate (150 ml, 1.32 mM) in a 250 ml three-neck round-flask equipped with a condenser was heated to boiling. After 5 min, tetrachloroauric acid (aq, 1.5 ml, 25 mM) were added. The solution turned light blue and then red. The solution was kept for 10 min on the heating element before allowing it to cool down under stirring at room temperature.

For the coating with different polymers, the NPs first had to be rendered hydrophobic. To this end, they were transferred to chloroform following the previous protocol using PEG for stabilization (PEGylation).[18] PEGylation is crucial to prevent aggregation during the phase transfer step. PEGylation was performed using mPEG-SH (M_w = 2000 g/mol), and PEG was added in a PEG to NP ratio of c_{PEG}/c_{NP} = $3x10^4$ to the NPs. The mixture was left stirring overnight at room temperature (RT). Then, the mixture was centrifuged at least three times whereby the NPs precipitated and the supernatant was replaced with fresh water, in order to obtain purified PEGylated Au NPs (PEG-Au NPs). To induce phase transfer, an excess of dodecylamine in chloroform (DDA, 0.4 M) was added to the PEG-Au NP solution (1:3 volume ratio chloroform:water) under vigorous stirring. After complete transfer of the PEG-Au NPs to the chloroform phase, the water phase was discarded. The NPs as transferred to chloroform were cleaned twice by centrifugation (i.e. precipitation and replacement of the supernatant with fresh chloroform) to remove excess unbound DDA and PEG molecules. The NP pellet was then redissolved in chloroform and kept for further experiments.

S2.2. Synthesis of Au NPs (5 nm).

Hydrophobic Au NPs were synthesized following the Brust-Schiffrin two-phase protocol with slight modifications according to previous reports.[18, 50] 300 mg of tetrachloroauric acid were dissolved in 25 ml Milli-Q water for the aqueous phase. The organic phase contained 2.170 g tetraoctylammonium bromide (TOAB) dissolved in 80 ml of toluene. The solutions were mixed in a 500 ml separation funnel and shaken vigorously for about 5 minutes. When the organic phase had turned deep orange and the initial yellowish color of the aqueous phase had disappeared (indicating complete transfer of gold ions to the organic phase), the aqueous phase was discarded and the organic phase was transferred to a 250 ml round flask. Then, aqueous sodium borohydride (25 ml, 353 mM) was added to the organic phase under vigorous stirring. The solution turned from deep orange to red-violet, indicating the formation of TOAB capped Au NPs, here termed as TOAB-Au NPs. This solution was stirred for 1 h to allow for reduction of the remaining gold ions. Afterwards, the solution was transferred to a separation funnel to discard the aqueous phase. The organic phase was washed with 25 ml of 10 mM HCl, 25 ml of 10 mM NaOH, and 3 times with 25 ml water. The remaining organic solution (ca. 80 ml) was transferred to a 250 ml round flask and stirred overnight to improve the size distribution.

Ligand exchange in order to warrant for a more stable coating was achieved with established protocols.[18] To replace TOAB with dodecanethiol (DDT), 10 ml of DDT were added to the TOAB-Au NP solution. The mixture was heated to 65 °C and stirred for 2 h. Then it was stirred without heating until

it had cooled down to RT to obtain dodecanethiol-stabilized Au NPs (DDT-Au NPs). The solution of DDT-Au NPs was centrifuged (900 g, 5 min) to remove large agglomerates. The supernatant was collected and the remaining DDT-Au NPs were precipitated with methanol. To this end, methanol was gradually added to aliquots (20 ml) of DDT-Au NP solutions until the solution turned cloudy. The solution was then centrifuged (900 g, 5 min) and the clear supernatant was replaced with chloroform. This purification step was repeated. In the second purification step a larger amount of methanol has to be added until the solution turns cloudy. The collected DDT-Au NPs were then suspended in chloroform.

S3. Amphiphilic polymers used in the present study.

The synthesis of poly(isobutylene-*alt*-maleic anhydride)-*graft*-dodecylamine (PMA-*g*-DDA) was done as reported previously.[6, 18, 21, 86-87] DDA (2.70 g, 14.5 mmol) in THF (100 ml) was added to 3.084 g PMA (3.084 g, 5.1 mmol) in a 250 ml flask and sonicated for 20 s, then heated to 55-60 ^{o}C for 3 h under stirring. The mixture turned transparent indicating the coupling of DDA to the PMA anhydride rings. The solution was concentrated to 40 ml by rotary evaporation and left overnight under reflux and stirring. Then, it was dried using a rotary evaporator and the obtained residue was dissolved in 40 ml of chloroform, yielding a 0.5 M monomer concentration. The other polymers were synthesized according to previous protocols.[47, 68, 82]

S4. Polymer coating of the nanoparticles.

The polymer coating process was carried out according to established protocols.[6, 18, 21, 68, 82] The solution of the amphiphilic polymer as dissolved in chloroform at a monomer concentration of c_p = 0.5 M was added to the NP solution. The needed volume of the polymer solution V_p was calculated according to:

$$V_p = \frac{R \times A_{eff} \times c_{NP} \times V_{NP}}{c_p} \qquad (1)$$

Where, V_p is the volume of polymer stock solution, R is the number of monomer units to be added per nm^2 of the effective surface area, $A_{eff} = \pi \times d_{eff}^{2}$ is the effective surface area of the NPs with $d_{eff} = d_c + 2l_{ligand}$ (d_c = core diameter, l_{ligand} = length of capping ligand). c_{NP} and V_{NP} are the concentration and volume of the NP dispersion, respectively, and c_p is the concentration of the stock polymer solution in terms of monomer concentration, which in our case was c_p = 0.5 M. As coating density R = 50 nm^{-2} was chosen for the case of the QDs and Au NPs (5 nm NP), and R = 2000 nm^{-2} in the case of Au NPs (17 nm).

In the case of the QDs, the concentration $c_{NP} = c_{QDs}$ was determined by using the molar extinction coefficient (at 350 nm) $\varepsilon_{350} = 1.5 \cdot 10^6\ M^{-1}cm^{-1}$. In the case of the Au NPs the concentration c_{NP} was determined by detection the concentration of elemental Au divided by the number of Au atoms per Au NP (which was derived by the mass of one Au NP (as given by the volume of one NP determined by the core diameter d_c as obtained from the TEM measurements times the density of bulk gold) divided by the mass of one gold atom).[18]

The mixture was then stirred at the rotary evaporator for 10 min, before evaporating the chloroform slowly under reduced pressure. Then a basic buffer was added for samples coated with zwitterionic and anionic polymers and an acidic buffer for samples coated by cationic polymers. SBB pH 12 (sodium borate 50 mM, pH 12) was used as basic buffer and diluted 0.1 M HCl at pH 3.3 was used as acidic "buffer". To remove large agglomerates, the sample was filtered by syringe filtration (0.22 µm, syringe filter, PTFE) and centrifuged 3x times to remove empty micelles, i.e. the NPs were precipitated and empty polymer micelles remaining in the supernatant were discarded.

S5. Characterization of the polymer-coated gold nanoparticles.

S5.1. UV-vis absorption spectroscopy.

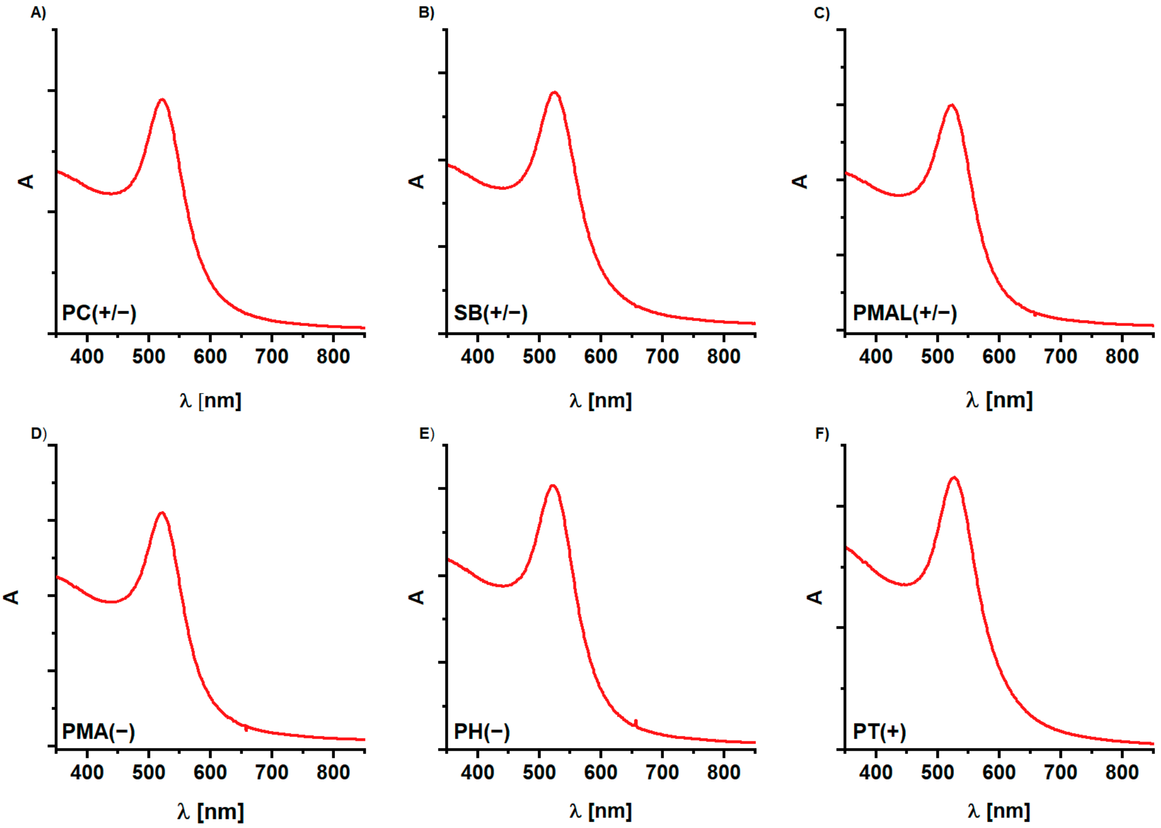

Figure S1. UV/vis absorption spectra of Au NPs (17 nm) as coated with A) PC(+/-), B) SB(+/-), C) PMAL(+/-), D) PMA(-), E) PH(-), and F) PT(+).

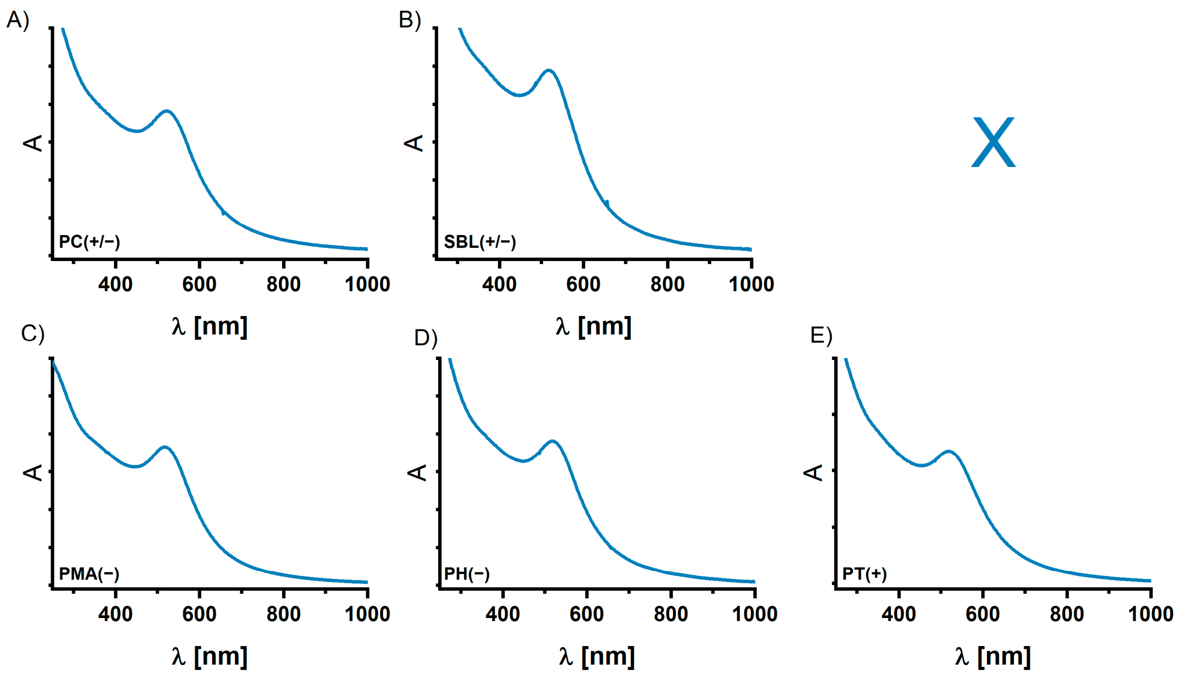

Figure S2. UV/vis absorption spectra for Au NPs (5 nm) coated with A) PC(+/-), B) SBL(+/-), C) PMA(-), D) PH(-), and E) PT(+).

S5.2. Dynamic Light Scattering (DLS).

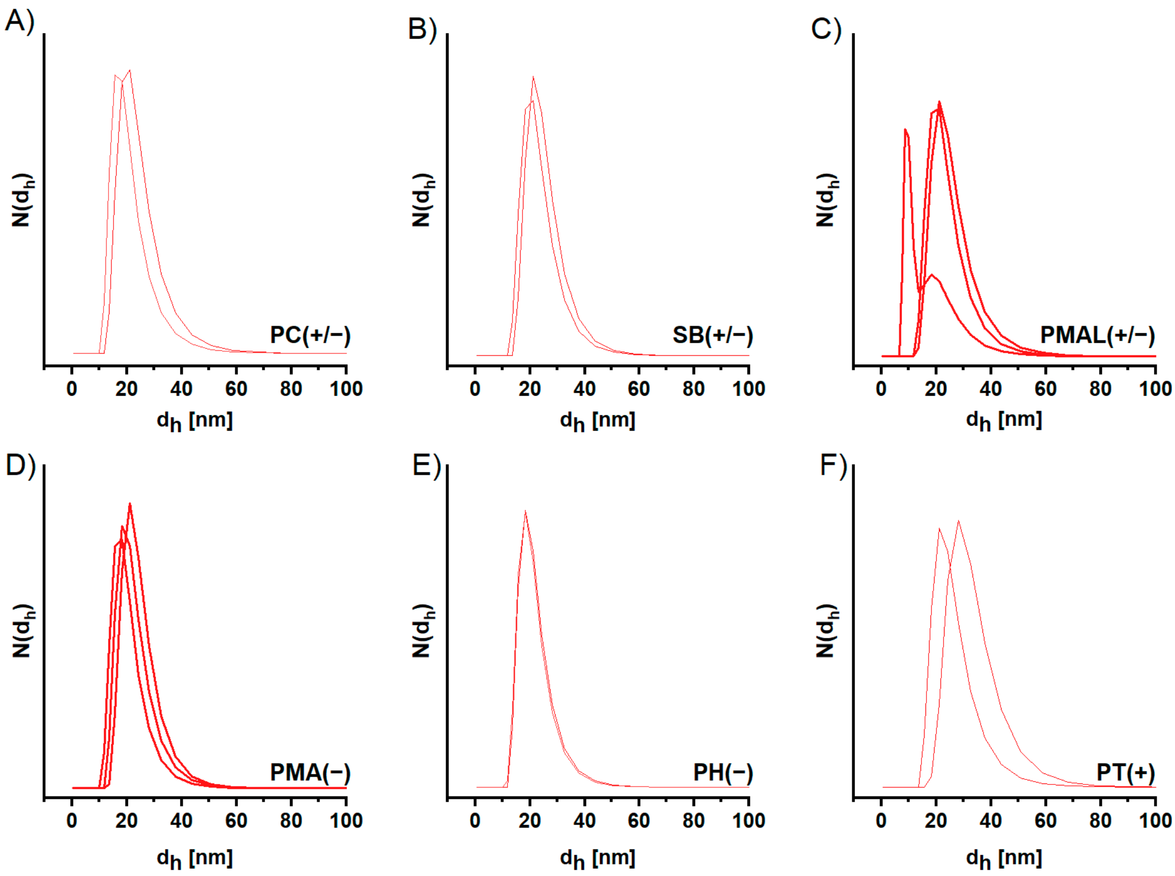

Figure S3. Number-weighted distributions *N(dₕ)* of the hydrodynamic diameters d_h of Au NPs (17 nm) coated with A) PC(+/-), B) SB(+/-), C) PMAL(+/-), D) PMA(-), E) PH(-), and F) PT(+) as obtained by DLS measurements in water.

Table S1. Summarized characterization of Au NPs (17 nm) coated with different polymers. d_h = mean hydrodynamic diameter as determined from DLS number-weighted size distribution. ζ = zeta potential. The indicated errors are the standard deviations of the mean of at least three measurements. λ_{SPR} = wavelength of the surface plasmon resonance peak as measured by UV-Vis absorption spectroscopy, and $\Delta\lambda_{FWHM}$ is the according full width at half maximum.

sample	formal charge	d_h [nm]	ζ [mV]	λ_{SPR} [nm]	$\Delta\lambda_{FWHM}$ [nm]
Au NPs@PC	+/-	21±2	7±2	521	46
Au NPs@SB	+/-	23±1	12±3	524	50
Au NPs@SBL	+/-	23±1	-32±3	524	50
Au NPs@PMAL	+/-	20±5	4±1	524	48
Au NPs@PMA	-	21±2	-52±5	520	45
Au NPs@PH	-	20±1	-42±1	521	47
Au NPs@PT	+	28±4	48±3	527	50
Au NPs@PTL	+	25±1	36±1	529	52

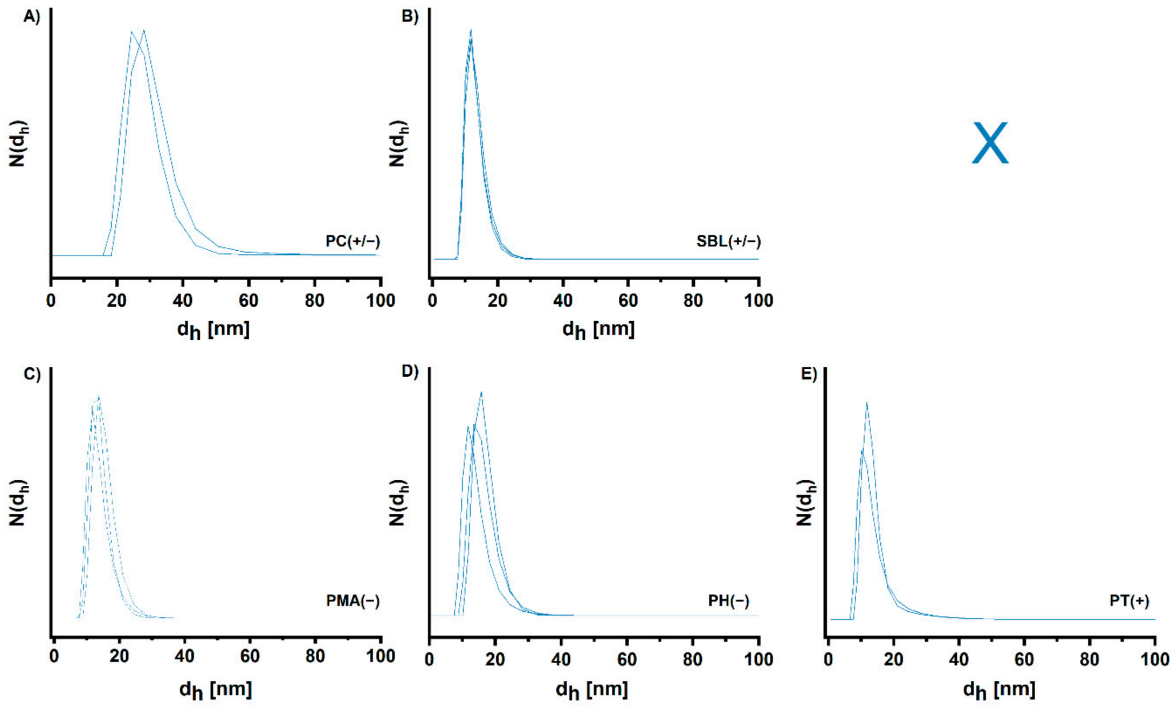

Figure S4. Number-weighted distributions $N(d_h)$ of the hydrodynamic diameters d_h of Au NPs (5 nm) coated with A) PC(+/-), B) SBL(+/-), C) PMA(-), D) PH(-), and E) PT(+) as measured by DLS.

Table S2. Summarized characterization of Au NPs (5 nm) coated with different polymers. d_h = mean hydrodynamic diameter as determined from DLS number-weighted size distribution. ζ = zeta potential. The indicated errors are the standard deviations of the mean of at least three measurements. λ_{SPR} = wavelength of the surface plasmon resonance peak as measured by UV-Vis absorption spectroscopy, and $\Delta\lambda_{\mathrm{FWHM}}$ is the according full width at half maximum.

sample	formal charge	d_{h} [nm]	ζ [mV]	λ_{SPR} [nm]
Au NPs@PC	+/-	30±2	-5±0.2	522
Au NPs@SBL	+/-	12±3	-25±2	517
Au NPs@PMA	-	15±1	-36±3	520
Au NPs@PH	-	13±1	-28±2	518
Au NPs@PT	+	13±1	13±1	525

S6. Characterization of polymer-coated QDs.

S6.1. UV-vis absorption and fluorescence spectroscopy.

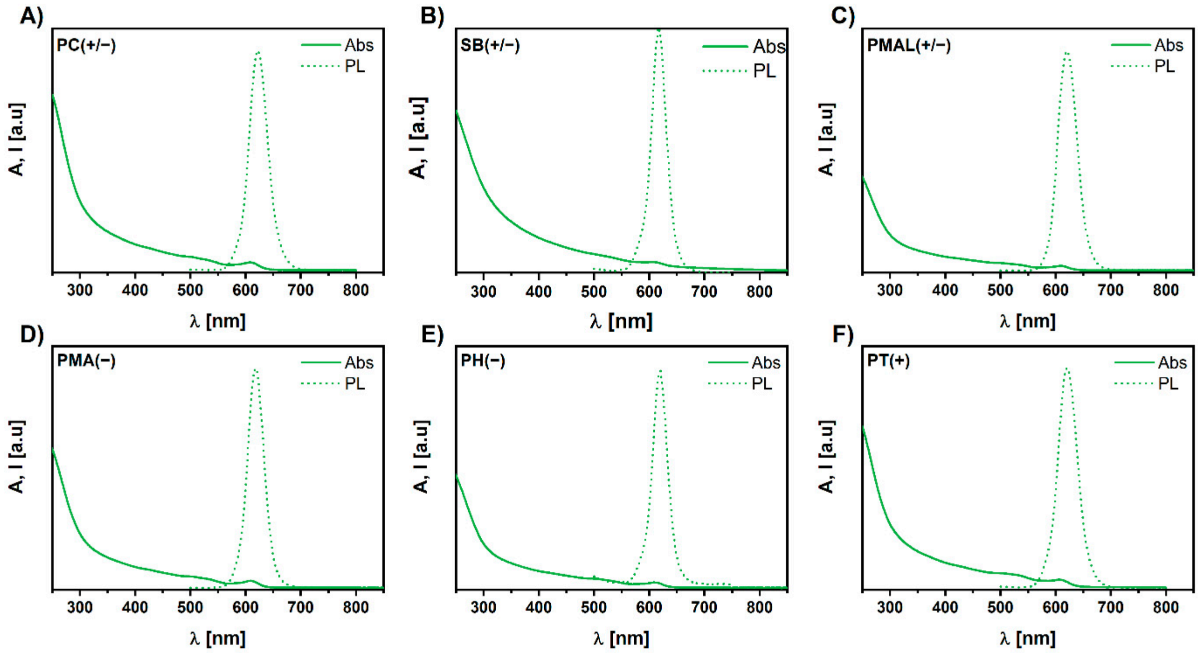

Figure S5. UV-Vis absorption *A(λ)* and photoluminescence (PL) spectra *I(λ)* of QDs as coated with A) PC(+/-), B) SB(+/-), C) PMAL(+/-), D) PMA(-), E) PH(-), and F) PT(+).

S6.2. Dynamic Light Scattering (DLS).

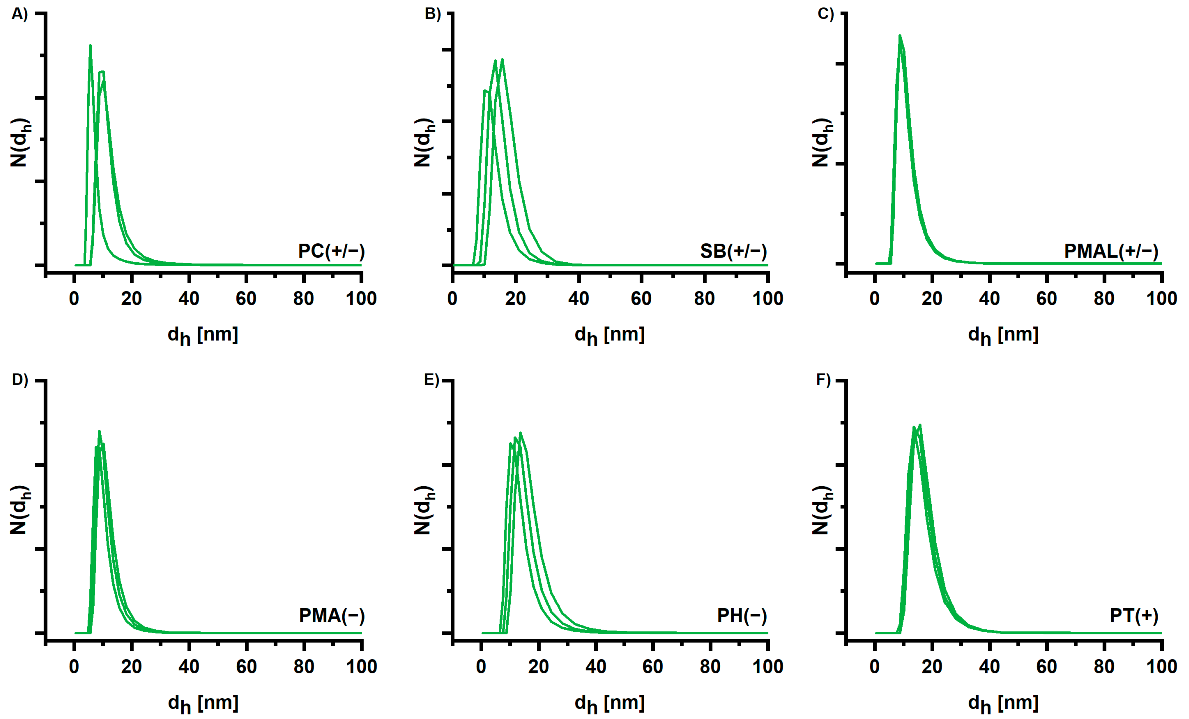

Figure S6. Number-weighted distributions $N(d_h)$ of hydrodynamic diameters d_h of *QDs* coated with A) PC(+/-), B) SB(+/-), C) PMAL(+/-), D) PMA(-), E) PH(-), and F) PT(+).

Table S3. Summarized characterization of QDs coated with different polymers. $r_h(DLS)$ = mean hydrodynamic radius as determined from DLS number-weighted size distribution. The indicated errors are the standard deviations of the mean of at least three measurements. $r_h(FCS)$ = mean hydrodynamic radius as determined from FCS measurements (see Table S6). ζ = zeta potential. The indicated errors are the standard deviations of the mean of at least three measurements. λ_{SPR} = wavelength of the surface plasmon resonance peak as measured by UV-Vis absorption spectroscopy, and $\Delta\lambda_{FWHM}$ is the according full width at half maximum.

sample	formal charge	r_h (DLS) [nm]	r_h (FCS) [nm	ζ [mV]
QDs@PC	+/-	6±0.6	8.5±0.2	-1±0.1
QDs@SB	+/-	7±1	12±0.5	-22±1
QDs@PMAL	+/-	5±0.2	8.4±0.2	4±1
QDs@PMA	-	5±0.4	6.9±0.1	-11±3
QDs@PH	-	7±0.9	7±0.4	-42±4
QDs@PT	+	8±0.3	7±0.3	44±1.5

S7. Colloidal stability of the NPs in NaCl solutions.

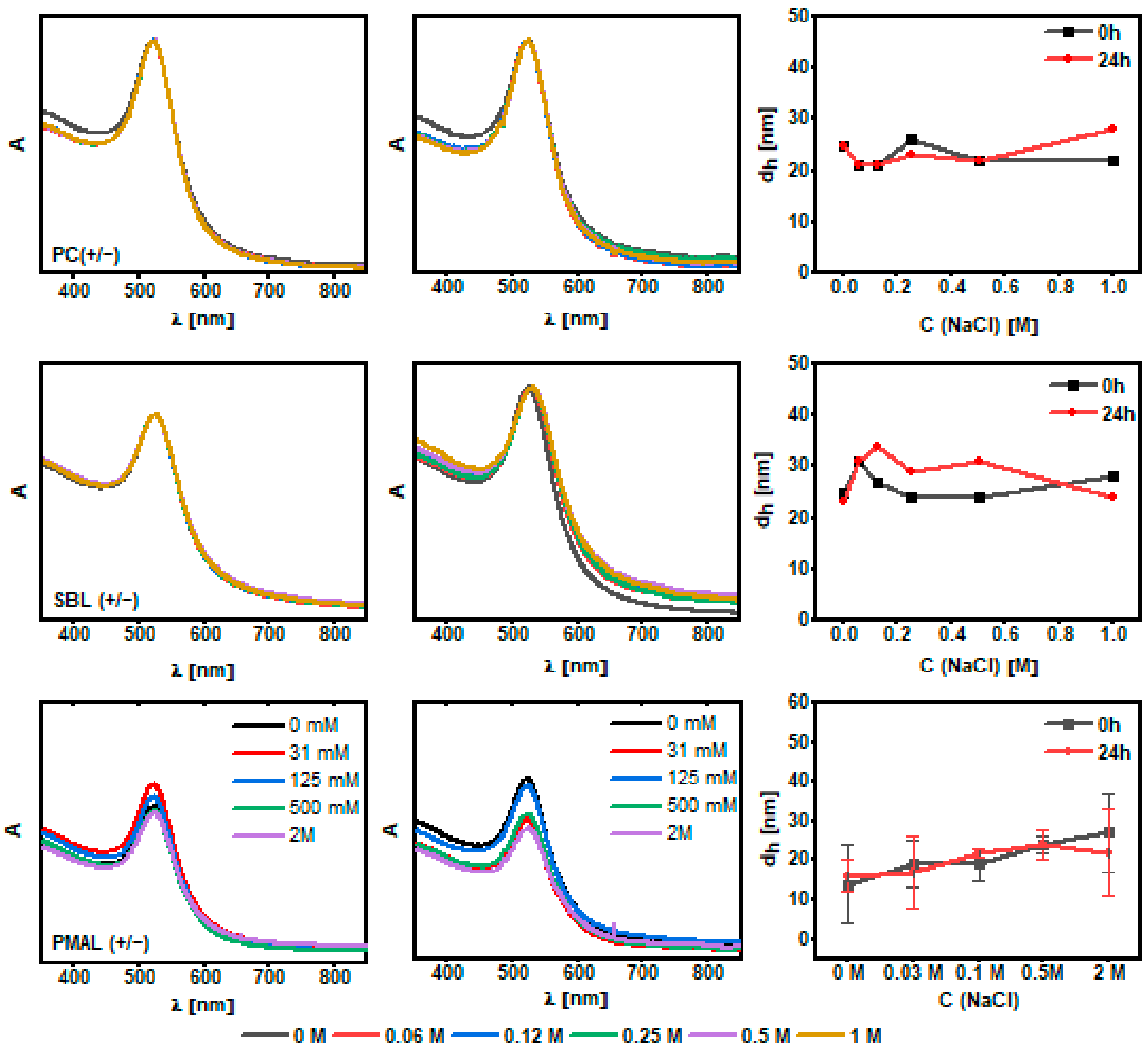

Figure S7. Probing of the colloidal stability of zwitterionic conjugates in aqueous solutions with different salt concentrations c(NaCl). From the top to the bottom row the results for Au NPs (17 nm) coated with PC(+/-), SBL(+/-), and PMAL(+/-) are presented. Left and middle column: UV-Vis absorption spectra A(λ) at different salt concentrations c(NaCl) directly after NaCl addition (0 h, left) and after 24 h (right). Right column: hydrodynamic diameters (d_h) measured by DLS in the presence of different NaCl concentrations as indicated for the same conjugates after different exposure times *t*.

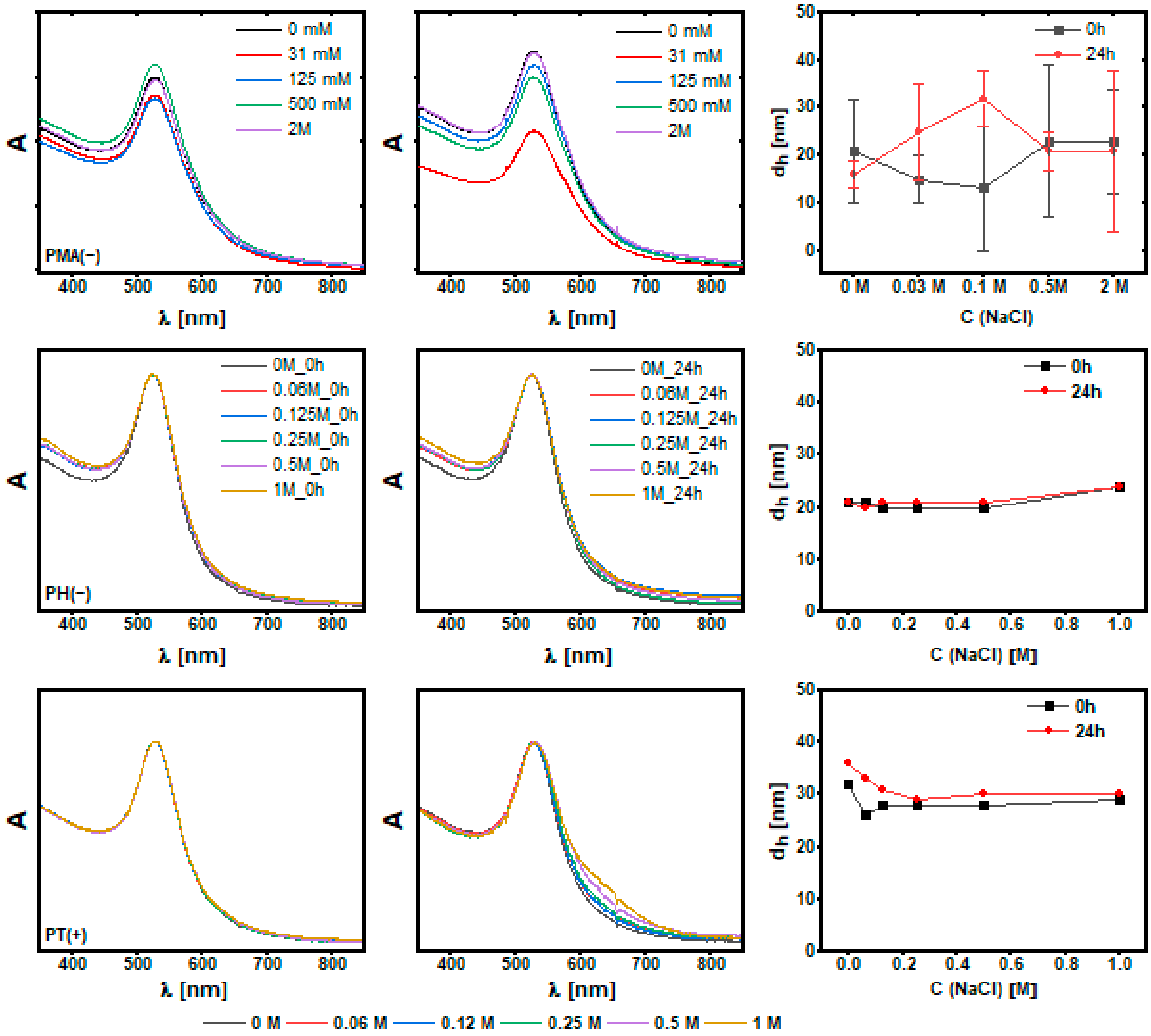

Figure S8. Probing of the colloidal stability of anionic and cationic conjugates in aqueous solutions with different salt concentrations c(NaCl). From the top to the bottom row the results for Au NPs (17 nm) coated with PMA(-), PH(-), and PT(+) are presented. Left and middle column: UV-Vis absorption spectra A(λ) at different salt concentrations c(NaCl) directly after NaCl addition (0 h, left) and after 24 h (right). Right column: hydrodynamic diameters (d_h) measured by DLS in the presence of different NaCl concentrations as indicated for the same conjugates after different exposure times *t*.

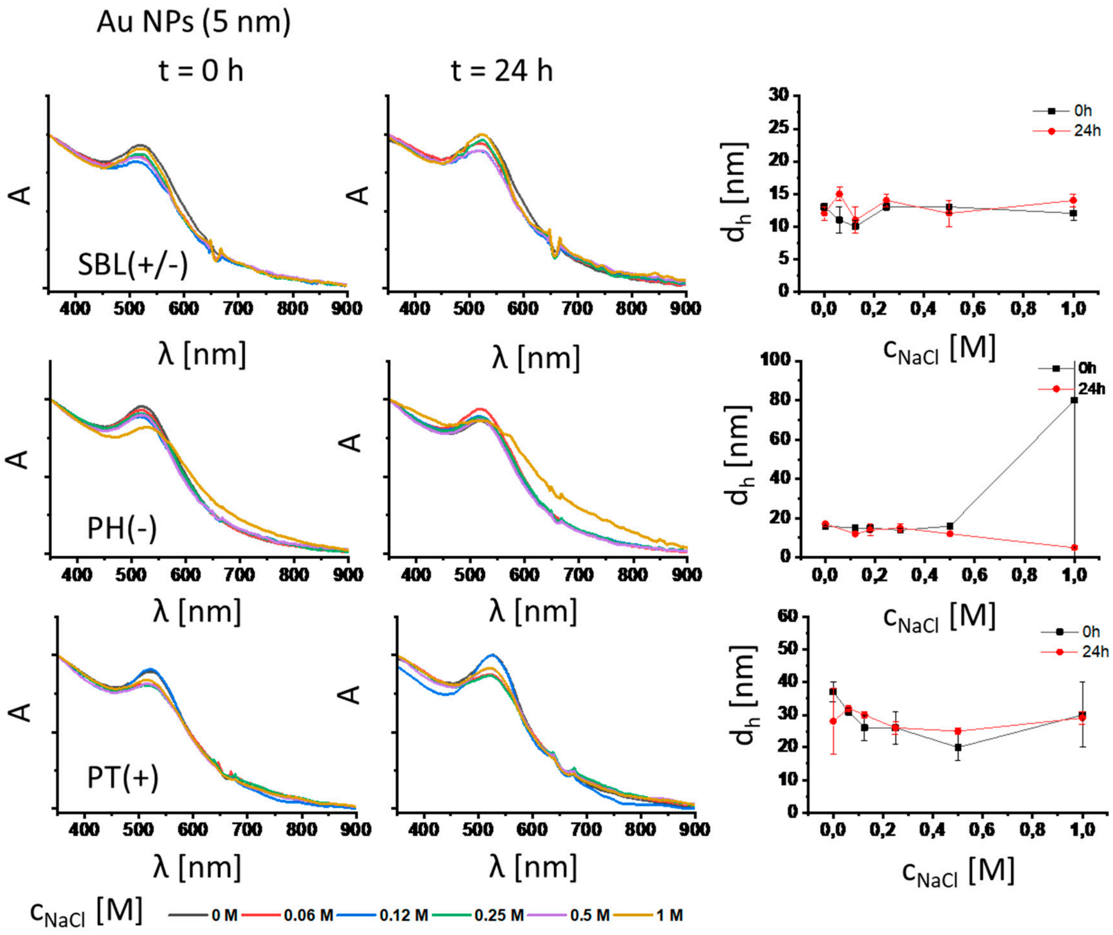

Figure S9. Probing of the colloidal stability of conjugates in aqueous solutions with different salt concentrations c(NaCl). From the top to the bottom row the results for Au NPs (5 nm) coated with SBL(+/-), PT(+), and PH(-) are presented. Left and middle column: UV-Vis absorption spectra A(λ) at different salt concentrations *c*(NaCl) directly after NaCl addition (0 h, left) and after 24 h (right). Right column: hydrodynamic diameters (d_h) measured by DLS in the presence of different NaCl concentrations as indicated for the same conjugates after different exposure times *t*.

S8. Dynamical interfacial tension (IFT) measurements.

To evaluate the surface activity of the nanoconjugates, we measured the dynamics of the interfacial tension of the NPs with the pendant drop method using a Drop Shape Analysis system (DSA30S, Krüss, Germany).[88] The measurements were carried out analyzing the toluene-water interface by using a syringe plugged into a stainless-steel needle with a diameter of 1.85 mm. Briefly, a small drop of ca. 50 µl water is created in the toluene pool and the drop profile recorded as a function of time using a high frame rate camera. The interfacial tension γ_t was determined by analyzing the drop profile with the Young-Laplace model.[59, 62, 89]

The time dependence of the interfacial tension γ_t was fitted with the empirical Hua and Rosen equation.[62]

$$\gamma_t = \gamma_m + \frac{\gamma_o - \gamma_m}{1 + \left(\frac{t}{t_h}\right)^n} \qquad (2)$$

Here γ_t is the interfacial tension at time t, γ_o is the interfacial tension of the pure solvents used (water-toluene), γ_m is the interfacial tension at the meso-equilibrium, *n* is a dimensionless exponent, and t_h is the half-life time to reach γ_m.

The dynamics of the interfacial tension may be divided into four different regions, induction, the rapid fall, meso-equilibrium, and equilibrium. The first three regions can be fitted with the Hua-Rosen equation.[59, 62, 90]

In addition to that, by differentiating the Hua-Rosen equation with respect to time, the maximum surface tension decay rate v_{max} can be obtained.[59] It provides information about how fast the mesoequilibrium is reached, i.e. it reflects the dynamics of diffusion and assembly of the according nanoconjugates at the liquid-liquid interface.

$$v_{max} = \frac{n(\gamma_0 - \gamma_m)}{4\, t_h} \qquad (3)$$

The experimentally obtained values for the QDs are presented in Figure 5 of the main manuscript and Table S4. The experimentally obtained values for the Au NPs (17 nm) are presented in Figure S10 and Table S5.

Table S4. Fitting results for dynamic interfacial tension measurements of polymer-coated QDs. γ_m is the meso-equilibrium interfacial tension, t_h is the half-life time to reach this value, γ_{eq} is the equivalent interfacial tension as measured at the end time point, n is exponent constant, v_{max} is the decay rate of the interfacial tension. Very small standard deviations (SD) are not shown.

Sample	γ_m [mN/m]	t_h [s]	n	v_{max} [mN/ms] *10^{-3}	γ_{eq} [mN/m]
QDs@PC (+/-)	16.5±0.01	295±0.5	1.1	18	16.2±0.7
QDs@SB (+/-)	24±0.5	19.4±4	0.55±0.07	85.4±4	24.3±0.7
QDs@PMAL (+/-)	12.2±1.5	593±182	0.5±0.03	5±1.7	16.8±1.3
QDs@PMA (-)	----	-----	-----	------	32.3±0.5
QDs@PH (-)	----	-----	-----	------	32.3±0.4
QDs@PT (+)	----	-----	-----	------	33.6±0.1

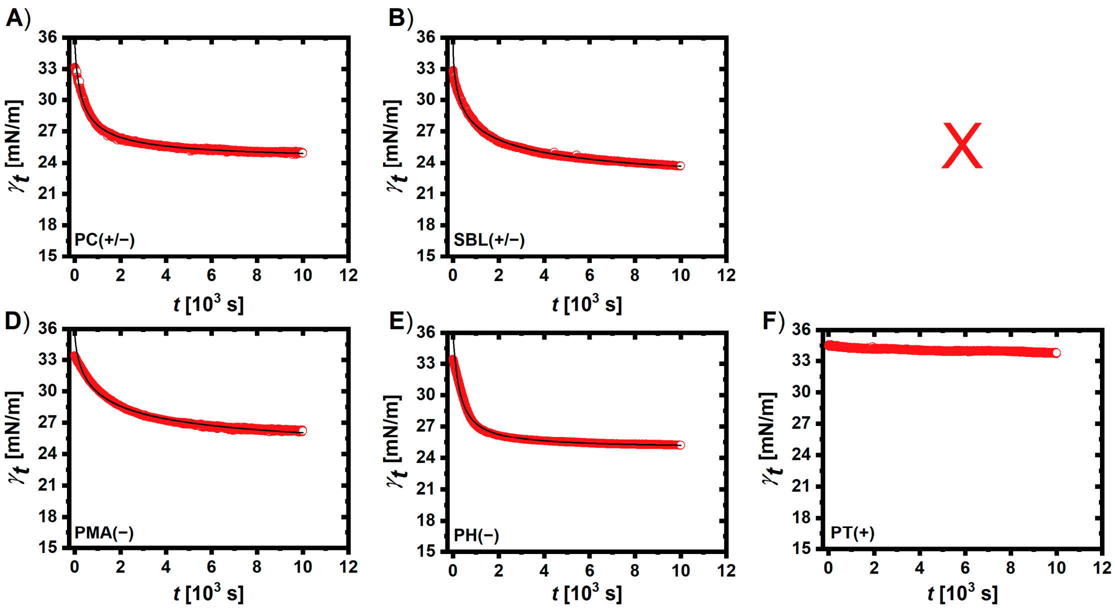

Figure S10. Interfacial tension γ_t (water-toluene) as function of time, as modulated by polymer-coated Au NPs dispersed in the water phase. The Au NPs (17 nm) were coated with A) PC(+/-), B) SBL(+/-), C) PMA(-), D) PH(-), and E) PT(+). The black lines are fits with the Hua and Rosen equation.

Table S5. Fitting results for dynamic interfacial tension measurements of polymer-coated Au NPs (17 nm). $\boldsymbol{\gamma_m}$ is the meso-equilibrium interfacial tension, $\boldsymbol{t_h}$ is the half-life time to reach this value, $\boldsymbol{\gamma_{eq}}$ is the equivalent interfacial tension as measured at the end time point, $\boldsymbol{n}$ is exponent constant, $\boldsymbol{v_{max}}$ is the decay rate of the interfacial tension. Very small standard deviations (SD) are not shown.

Sample	γ_m *[mN/m]*	t_h *[s]*	*n*	$\boldsymbol{v_{max}}$ *[mN/ms] *10^{-3}*	γ_{eq} *[mN/m]*
Au NPs@PMA (-)	23.7±0.3	1513±599	0.6	1.3±0.5	26.8±0.8
Au NPs@PH (-)	25±0.5	303±25	1	9.4±0.6	25±0.3
Au NPs@PT (+)	---	----	----	----	32.8±1.3
Au NPs@PTL (+)	---	----	----	----	32.7±0.3
Au NPs@PC (+/-)	23.8±0.3	295±42	0.76	7.8±1	24.65±0.3
Au NPs@SBL (+/-)	21.3±1.9	794±95	0.5	2.4±0.3	24±1.1

S9. Fluorescence correlation spectroscopy (FCS) measurements.

The protein adsorption on the QDs was measured with FCS using a confocal light scanning Microscopy (CLSM) (LSM 880, Zeiss, Germany) with a Zeiss Plan-Apochromat 40x/1.0 water (WD 2.5 mm) objective. Human serum albumin (HSA) and human transferrin (Tf) were mixed with a fixed concentration of the QDs (c_{QD} = 50 nM) in a phosphate-buffered saline solution (PBS). The solutions of the protein were varied in concentration from c_p = 0 µM to 500 µM prepared by a sequential dilution of a concentrated stock solution. All samples were incubated for 15 min before carrying out the measurements. The samples were added into a 35 mm petri dish with a glass bottom, were then flipped upside down and the sample was kept in place using a glass coverslip (0.17 ± 0.005 mm thickness). Each measurement was collected for ~ 8 min with three independent measurements for each data point. The focal volume (with radius r_f) was calibrated at a 488 nm laser line using rhodamine 6G with the known diffusion coefficient $D_{R6G} = 414 \pm 1 \frac{\mu m^2}{s}$ to calibrate the width of the focal volume (Gaussian ellipsoid approximation) as shown in equation (4). With the focal volume, the diffusion time τ_D measured by FCS for the QD samples can be related to the corresponding diffusion coefficient *D*.[63-64]

$$\tau_D = \frac{r_f^2}{4\,D} \tag{4}$$

τ_D is the diffusion time, r_f is the radius of the effective focal volume and *D* is the diffusion coefficient.

The diffusion coefficient D was obtained by fitting the FCS data using the FCS module implemented with the ZEN software from Zeiss, according to equation (5)

$$G(\tau) = \frac{1}{N_{NP}}\left(1 + \frac{T}{1-T}\, e^{-\tau/\tau_T}\right)\sum_{i=1}^{M}\frac{f_i}{1+ {}^{\tau}/_{\tau_{Di}}}\,\frac{1}{\sqrt{1+ {}^{\tau}/_{\tau_{Di}}\, S^2}} \tag{5}$$

Here N_{NP} is the number of the NPs within the effective focal volume, *M* is the fraction of the individual components (here just one component, the NPs, i.e. *M* = 1), τ_{Di} is the diffusion time, *S* is the ratio of the axes of the effective focal volume, *T* is the fraction of the fluorescence decay from the triplet state of the sample and τ_T is the triplet state lifetime. In case of the QDs as investigated here we have excluded the triplet state contribution from the fitting equation, as we expect no significant blinking at low excitation power for the core-shell-shell QD we are using. This leads to the following simplified equation.

$$G(\tau) = \frac{1}{N_{NP}}\,\frac{1}{1+ {}^{\tau}/_{\tau_D}}\,\frac{1}{\sqrt{1+ {}^{\tau}/_{\tau_D}\, S^2}} \tag{6}$$

The hydrodynamic radius was calculated according to the Stokes-Einstein relation using equations 6 and 4:

$$r_h = \frac{k_B\,T}{6\pi\eta D} \tag{7}$$

Here k_B is the Boltzmann constant, η is the dynamic viscosity of the solvent, T is the absolute temperature and D is the diffusion coefficient. The protein effect on the solution's viscosity was corrected with the intrinsic viscosity of the proteins assuming a linear dependence $\eta = (\eta_i C_P + 1)\eta_o$, where η_i is the intrinsic viscosity of the according protein (4.13 cm^3/g for HSA and 4.4 cm^3/g for Tf), C_P is the protein concentration in g/cm^3, and η_o is the pure solvent's viscosity.

The dependency of the hydrodynamic radius of the protein concentration was fitted according to the following equation as described by Röcker et al.[63]:

$$r_h = r_h(0)\sqrt[3]{1 + cN} \tag{8}$$

Here, $r_h(0)$ is the hydrodynamic radius of the bare polymer coated QDs without adsorbed proteins, $(r_p/r_h(0))^3$ is the ratio of the volume of one protein (with radius r_p) and the volume of one QD (with radius $r_h(0)$) and N_p is the number of protein molecules adsorbed per QD. The dependency of N_p from the protein concentration was fitted using the Hill model according to:

$$N_p = N_{p,max} \frac{1}{1 + (K_D/c_p)^n} \tag{9}$$

Where $N_{p,max}$ is the maximum possible number of proteins bound per QD at saturation, K_D is the dissociation constant, c_p is the protein concentration (here human serum albumin (HSA) or transferrin (Tf)), and n is the Hill coefficient.[40, 63, 68, 91-94]

The fits for the experimentally obtained curves shown in Figure 6 and Figure 7 in the main article are provided in Tables S6 and S7. The structures of HSA and Tf are shown in Figure S11.

Table S6. Fitting parameters (Hill-model) for the FCS results as obtained for HSA adsorption to QDs. *$r_h(0)$* is the hydrodynamic radius of the QDs at 0 µM of HSA, $\boldsymbol{K_D}$ is the dissociation constant, quantifying the binding affinity of the proteins to the nanoconjugates (QDs + protein shell). *$(r_p/r_h(0))^3$* is the volume ratio of protein to QD, $\boldsymbol{n}$ is the Hill coefficient, $\boldsymbol{N_{p,max}}$ is the maximum number of proteins adsorbed to the surface of one QD at saturation, and $\Delta\boldsymbol{r_h}$ is the thickness of the protein layer ("corona") adsorbed to the QDs.

Sample	$r_h(0)$ [nm]	K_D [µM]	$(r_p/r_h(0))^3$	n	$N_{p,max}$	Δr_h [nm]
QDs@PMAL (+/-)	6.4±0.04	22±6	0.076	1.5±0.3	29±5	2.6±0.1
QDs@PMA (-)	6.88±0.09	20.8±8	0.05	0.9±0.2	40.3±7	2.9±0.1
QDs@PH (-)	6.6±0.06	183±109	0.076	0.76±0.08	106±26	5±0.1
QDs@PT (+)	7.14±0.07	7.5±2	0.067	2±1	16.5±3	1.9±0.1

Table S7. Fitting parameters (Hill-model) for the FCS results as obtained for Tf adsorption to QDs. *$r_h(0)$* is the hydrodynamic radius of the QDs at 0 µM of Tf, K_D is the dissociation constant, quantifying the binding affinity of the proteins to the nanoconjugates (QDs + protein shell). *$(r_p/r_h(0))^3$* is the volume ratio of protein to QD, n is the Hill coefficient, $N_{p,max}$ is the maximum number of proteins adsorbed to the surface of one QD at saturation, and Δr_h is the thickness of the protein layer ("corona") adsorbed to the QDs.

Sample	$r_h(0)$ [nm]	K_D [µM]	$(r_p/r_h(0))^3$	n	$N_{p,max}$	Δr_h [nm]
QDs@PMAL (+/-)	6.5±0.04	172±66	0.248	1.5±0.4	5.5±1.4	1.9±0.05
QDs@PMA (-)	5.79±0.1	35.6±7	0.38	1.33±0.2	16.9±2	5.2±0.2
QDs@PH (-)	6.7±0.2	115±85	0.233	0.8±0.2	38±12	6.5±0.23
QDs@PT (+)	7.27±0.08	35.5±9	0.197	1.6±0.3	10.9±2	3±0.08

The expected number of proteins to saturate the surface of the QD was estimated from the area ratio of the proteins to the QD surface area. The surface area of the QD was calculated with *$r_h(0)$*. The contact areas of the proteins were estimated assuming an equilateral triangular surface of 8 nm for HSA and an approximated rectangular surface of 8.4 x 5 nm^2 for Tf based on the structure of the according proteins, see Figure S11. Based on this estimation, ~ 21 HSA molecules and ~ 12 Tf molecules – on average – are needed to saturate the surface of one QD, while the expected monolayer thickness of the HSA is ~ 3 nm and for the Tf is ~7 nm.[93]

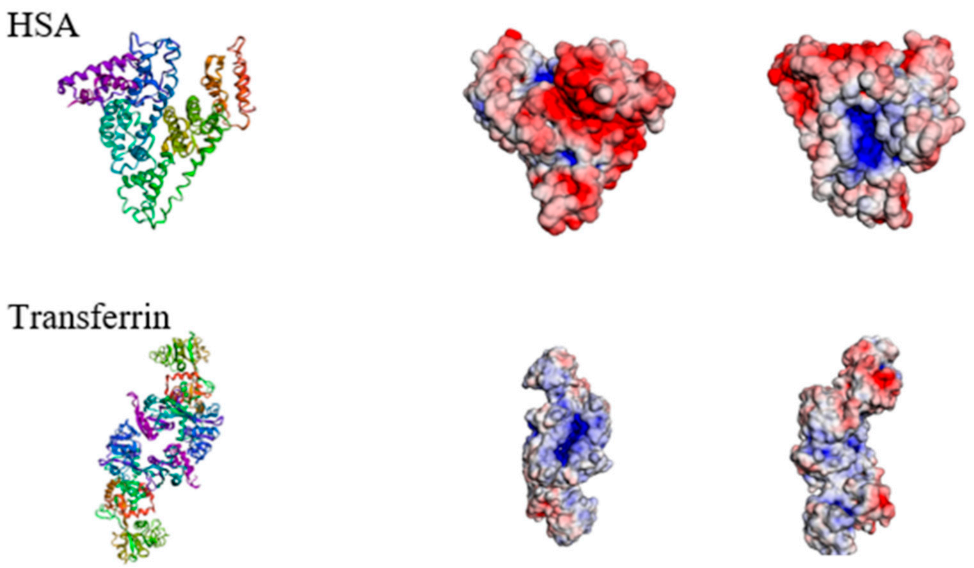

Figure S11. Surface electrostatic distribution of proteins. Left column: cartoon representation of HSA (PDB code: 1UOR), and Tf (PDB code: 2HAU). Right column: surface electrostatic calculated online via (https://server.poissonboltzmann.org/pdb2pqr) potential range +/- 5 k_BT/e at pH 7.4. The potential is represented in gradual color increasing from dark blue (most positive) to light blue, white, light red to dark red (most negative).

S10. Cell viability assays.

The biocompatibility of the NPs was examined with HeLa cells. HeLa cells were purchased from ATCC and were grown in DMEM medium supplemented with 10% fetal bovine serum (FBS) and 100 U/ml penicillin and streptomycin at 37 °C and 5% CO_2 until the desired confluence.

The cell viability of HeLa cells was evaluated by a resazurin assay.[40, 95] HeLa cells were seeded in 96-well plates at a density of 7500 cells/well in a 100 µl DMEM medium supplemented with 10% FBS per well. On the following day, the old medium was discarded and then a concentration series of the NPs in 100 µl (diluted in the same cell media) was exposed to the seeded cells for 24 h. After this exposure time, the NP solution was removed, and the cells were washed three times using PBS. Then, 100 ul of the resazurin working solution, 0.025 mg/ml, was added to each well and incubated for 4 h.

After the incubation time, the cell viability was estimated by measuring the fluorescence of each well with a fluorometer (Fluorolog-3, Horiba Jobin Yvon, USA) with excitation at 560 nm and emission from 580 to 590 nm. The cell viability V was calculated a fluorescence normalized to the fluorescence of cells which had not been exposed to NPs.[96]

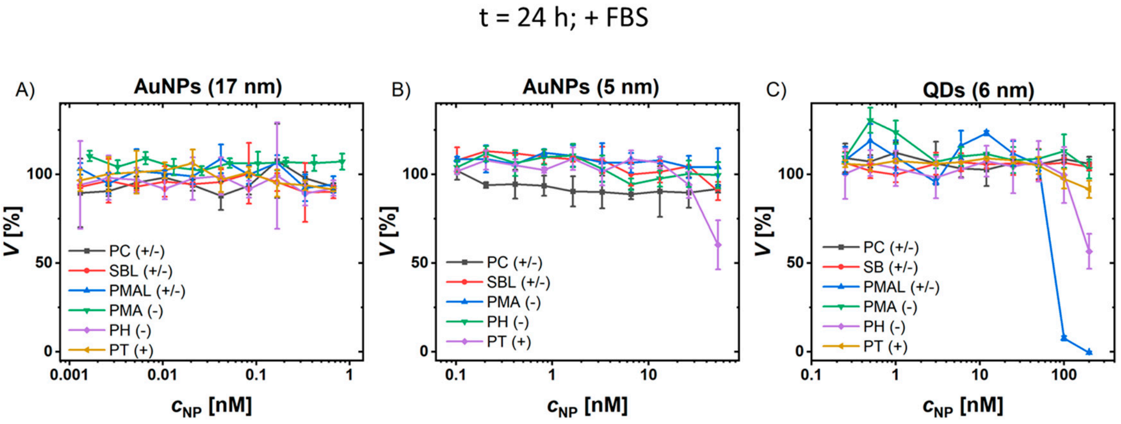

Figure S12. Cellular viability (V) of HeLa cells at different nanoparticle concentrations c_{NP}. A) Au NPs (17 nm), B) Au NPs (5 nm), C) QDs (6 nm). The data are the mean values from at least three independent measurements. Error bars indicate the standard deviation of the mean, some errors are too small to be discerned in the plot.

S11. NP uptake by cells.

S11.1. Inductively coupled plasma mass spectrometry (ICP-MS) quantification.

To quantify the cellular uptake of the (non-fluorescent) Au NPs, elemental analysis with ICP-MS was performed. The uptake was also tested with ICP-MS for some QD-conjugates to compare the results with those of the flow cytometry experiments.

To this end, HeLa cells were seeded in a 6-well plate at 200 x 10^3 cells/well for 24 h. Then, the cells were exposed to the desired concentration c_{NP} of NPs for 24 h. After incubation, the cells were washed three times with PBS and detached by adding 200 µl of 0.05% trypsin-EDTA. Cells were then collected in 1 ml PBS. The cells were counted (using a Neubauer chamber, Celeromics Technologies, Spain) and then centrifuged at 4000 rpm for 10 min.[40] To prepare the samples for elemental analysis, 75 µl of concentrated HNO_3 (67 %) were added to the cell pellets. The next day, 150 µl of concentrated HCl (37 %) were added to the same solution. After another 24 h, 2.275 ml HCl (2%) was added and the solution transferred to PFA tubes for ICP-MS measurements.

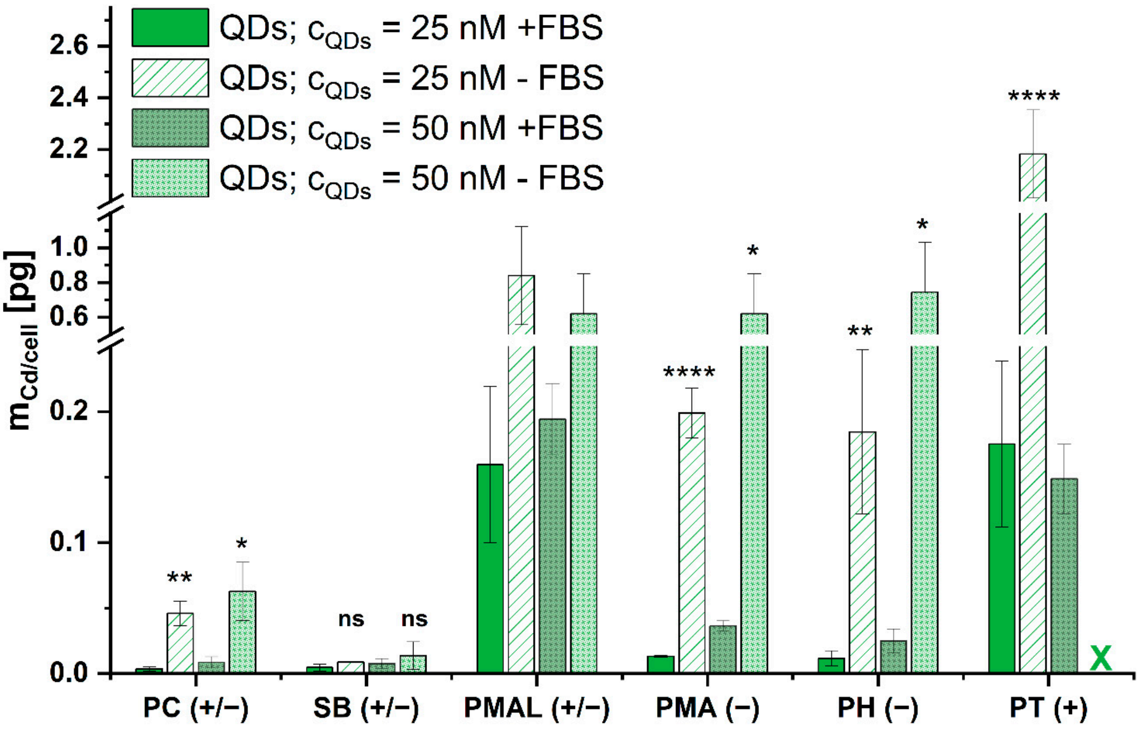

Figure S13. Cellular uptake of QDs by HeLa cells at 6 h exposure time at two different serum conditions: with (+ FBS) or without (- FBS) FBS supplement and different nanoparticle concentrations $c_{NP} = c_{QDs}$ as indicated. Data from at least three independent measurements are shown with the standard deviations of the mean. Significance tested with student's t-test (* P<0.05, ** P<0.01, and *** P<0.001).

S11.2. Flow cytometry quantification.

For assessing the cellular uptake of the QDs by flow cytometry, HeLa cells were seeded at 40000 cells/well at 1 ml per well in 24-well plates (Sarstedt, Germany) for 24 h. Then, the cells were washed and incubated with different concentrations c_{NP} of QDs. The cells were subsequently washed three times with PBS and detached from the well-plate with 0.05% trypsin-EDTA. The cells were collected by centrifugation at 300 g for 5 min and resuspended in 0.3 ml PBS for analysis with the flow cytometer (BD LSRFortessa^{TM}, BD Bioscience, US). The obtained results were analyzed with the software FlowJo_V10. The mean fluorescence intensity is presented after subtracting that of the control cells (which had not been exposed to QDs). The experiments were carried out in triplicates.

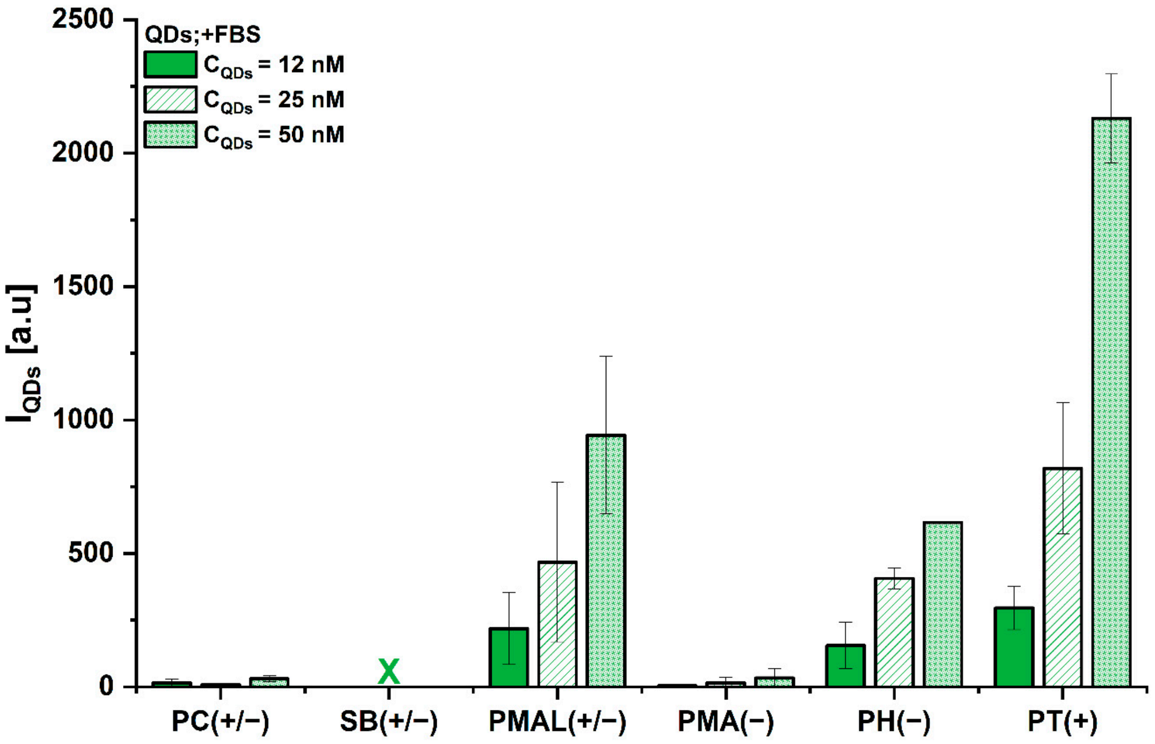

Figure S14. Cellular uptake of the QD in cell medium with 10% FBS after 24 h exposure time as measured by flow cytometry. The mean fluorescence intensity I_{QDs} (per cell; background corrected) of the cells with internalized QDs is shown. Data from at least three independent measurements are shown with the standard deviations of the mean.

S12. Control experiments with edelfosine.

As addition coating edelfosine (2-Methoxy-3-(octadecyloxy)propyl 2-(trimethylazaniumyl)ethyl phosphate, M_w = 523.73) was used (Sigma Aldrich SML0332). The physicochemical characterization is shown in Table S8. FCS data for the adsorption of HSA and Tf as shown in Figure S15B. These can be compared for data data obtained with the other polymers shown in Figures 6 and 7. In comparison to the other coatings the EDLF coated NPs seem to be more toxic (cf. Figure S15C versus Figure S12). The cellular uptake of the EDLF coated NPs is low (cf. Figure S15D versus Figure 8).

Table S8. Characterization of QDs coated with EDLF (+/-). r_h*(DLS)* = mean hydrodynamic radius as determined from DLS number-weighted size distribution. The indicated errors are the standard deviations of the mean of at least three measurements. r_h*(FCS)* = mean hydrodynamic radius as determined from FCS measurements. ζ = zeta potential. The indicated errors are the standard deviations of the mean of at least three measurements. For comparison the data of the other polymers are enlisted in Table S3.

sample	formal charge	r_h *(DLS) [nm]*	r_h *(FCS) [nm]*	ζ *[mV]*
QDs@EDLF	+/-	5.7±0.1	8±0.2	0.4±1

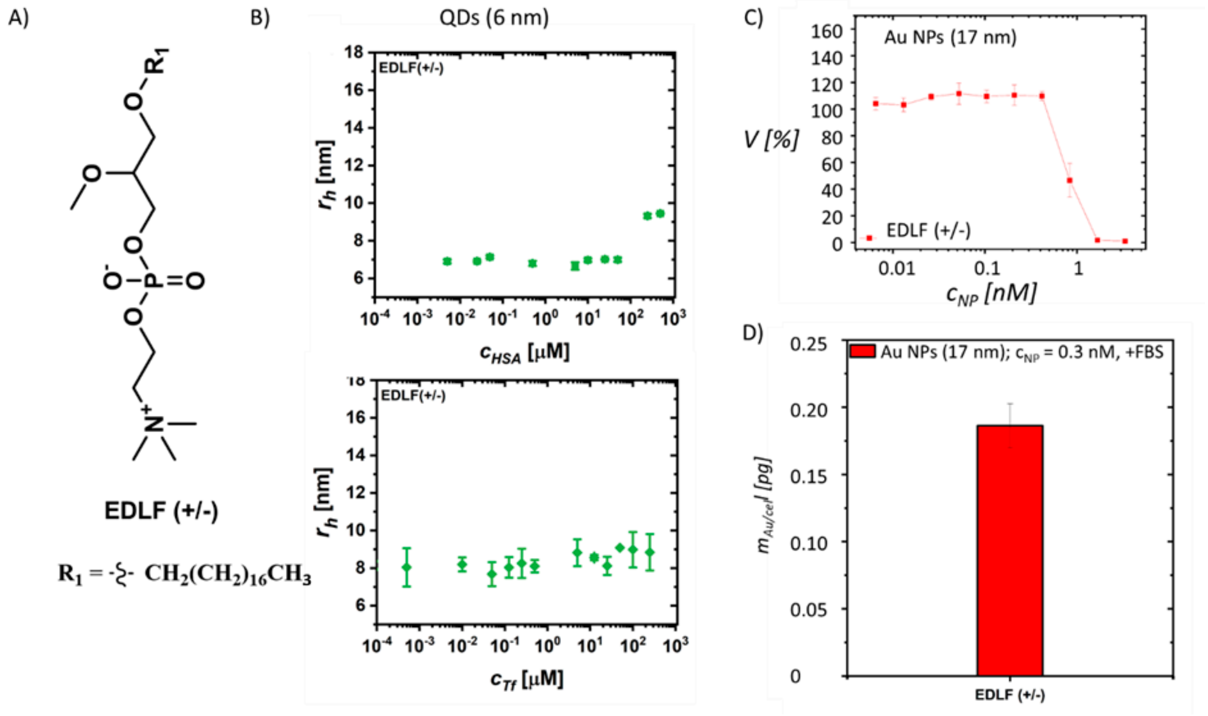

Figure S15. A) Structure formula of EDLF. B) Protein adsorption as quantified with FCS. C) Viability test. D) Uptake of NPs as studied with ICP-MS.

S13. References.

(same list as in main text)

1. Heuer-Jungemann, A.; Feliu, N.; Bakaimi, I.; Hamaly, M.; Alkilany, A.; Chakraborty, I.; Masood, A.; Casula, M. F.; Kostopoulou, A.; Oh, E.; Susumu, K.; Stewart, M. H.; Medintz, I. L.; Stratakis, E.; Parak, W. J.; Kanaras, A. G., The Role of Ligands in the Chemical Synthesis and Applications of Inorganic Nanoparticles. *Chem. Rev.* **2019,** *119*, 4819-4880.
2. Sapsford, K. E.; Algar, W. R.; Berti, L.; Gemmill, K. B.; Casey, B. J.; Oh, E.; Stewart, M. H.; Medintz, I. L., Functionalizing nanoparticles with biological molecules: developing chemistries that facilitate nanotechnology. *Chem. Rev.* **2013,** *113* (3), 1904-2074.
3. Jokerst, J.; Lobovkina, T.; Zare, R.; Gambhir, S., Nanoparticle PEGylation for imaging and therapy. *Nanomedicine-UK* **2011,** *6* (4), 715-728.
4. Mout, R.; Moyano, D. F.; Rana, S.; Rotello, V. M., Surface functionalization of nanoparticles for nanomedicine. *Chem. Soc. Rev.* **2012,** *41* (7), 2539-2544.
5. Chen, G.; Roy, I.; Yang, C.; Prasad, P. N., Nanochemistry and Nanomedicine for Nanoparticle-based Diagnostics and Therapy. *Chem. Rev.* **2016,** *116* (5), 2826-2885.
6. Zhang, F.; Lees, E.; Amin, F.; Rivera_Gil, P.; Yang, F.; Mulvaney, P.; Parak, W. J., Polymer-Coated Nanoparticles: A Universal Tool for Biolabelling Experiments. *Small* **2011,** *7*, 3113-3127.
7. Sperling, R. A.; Parak, W. J., Surface modification, functionalization and bioconjugation of colloidal inorganic nanoparticles. *Phil. Trans. R. Soc. A* **2010,** *368* (1915), 1333-1383.
8. Pöselt, E.; Schmidtke, C.; Fischer, S.; Peldschus, K.; Salamon, J.; Kloust, H.; Tran, H.; Pietsch, A.; Heine, M.; Adam, G.; Schumacher, U.; Wagener, C.; Förster, S.; Weller, H., Tailor-Made Quantum Dot and Iron Oxide Based Contrast Agents for in Vitro and in Vivo Tumor Imaging. *ACS Nano* **2012,** *6* (4), 3346-3355.
9. Stewart, M. H.; Susumu, K.; Mei, B. C.; Medintz, I. L.; Delehanty, J. B.; Blanco-Canosa, J. B.; Dawson, P. E.; Mattoussi, H., Multidentate Poly(ethylene glycol) Ligands Provide Colloidal Stability to Semiconductor and Metallic Nanocrystals in Extreme Conditions. *J. Am. Chem. Soc.* **2010,** *132* (28), 9804-9813.
10. Soliman, M. G.; B, B. P.; Parak, W. J.; Pino, P. d., Phase transfer and polymer coating methods toward improving the stability of metallic nanoparticles for biological applications. *Chem. Mater.* **2015,** *27*, 990-997.
11. Na, H. B.; Palui, G.; Rosenberg, J. T.; Ji, X.; Grant, S. C.; Mattoussi, H., Multidentate Catechol-Based Polyethylene Glycol Oligomers Provide Enhanced Stability and Biocompatibility to Iron Oxide Nanoparticles. *ACS Nano* **2012,** *6* (1), 389-399.
12. Palui, G.; Na, H. B.; Mattoussi, H., Poly(ethylene glycol)-Based Multidentate Oligomers for Biocompatible Semiconductor and Gold Nanocrystals. *Langmuir* **2012,** *28* (5), 2761-2772.
13. Schulz, F.; Dahl, G. T.; Besztejan, S.; Schroer, M. A.; Lehmkühler, F.; Grübel, G.; Vossmeyer, T.; Lange, H., Ligand Layer Engineering To Control Stability and Interfacial Properties of Nanoparticles. *Langmuir* **2016,** *32* (31), 7897-7907.
14. Sukhorukov, G. B.; Donath, E.; Lichtenfeld, H.; Knippel, E.; Knippel, M.; Budde, A.; Mohwald, H., Layer-by-layer self assembly of polyelectrolytes on colloidal particles. *Colloids Surf. A* **1998,** *137* (1-3), 253-266.
15. Donath, E.; Sukhorukov, G. B.; Caruso, F.; Davis, S. A.; Möhwald, H., Novel Hollow Polymer Shells by Colloid-Templated Assembly of Polyelectrolytes. *Angew. Chem. Int. Ed.* **1998,** *37* (16), 2202-2205.
16. Schmidtke, C.; Poselt, E.; Ostermann, J.; Pietsch, A.; Kloust, H.; Tran, H.; Schotten, T.; Bastus, N. G.; Eggers, R.; Weller, H., Amphiphilic, cross-linkable diblock copolymers for multifunctionalized nanoparticles as biological probes. *Nanoscale* **2013,** *5* (16), 7433-7444.
17. Ostermann, J.; Merkl, J. P.; Flessau, S.; Wolter, C.; Kornowksi, A.; Schmidtke, C.; Pietsch, A.; Kloust, H.; Feld, A.; Weller, H., Controlling the Physical and Biological Properties of Highly Fluorescent Aqueous Quantum Dots Using Block Copolymers of Different Size and Shape. *ACS Nano* **2013,** *7* (10), 9156-9167.

18. Hühn, J.; Carrillo-Carrion, C.; Soliman, M. G.; Pfeiffer, C.; Valdeperez, D.; Masood, A.; Chakraborty, I.; Zhu, L.; Gallego, M.; Zhao, Y.; Carril, M.; Feliu, N.; Escudero, A.; Alkilany, A. M.; Pelaz, B.; Pino, P. d.; Parak, W. J., Selected Standard Protocols for the Synthesis, Phase Transfer, and Characterization of Inorganic Colloidal Nanoparticles. *Chem. Mater.* **2017,** *29*, 399−461.
19. Schmidtke, C.; Eggers, R.; Zierold, R.; Feld, A.; Kloust, H.; Wolter, C.; Ostermann, J.; Merkl, J.-P.; Schotten, T.; Nielsch, K.; Weller, H., Polymer-Assisted Self-Assembly of Superparamagnetic Iron Oxide Nanoparticles into Well-Defined Clusters: Controlling the Collective Magnetic Properties. *Langmuir* **2014,** *30* (37), 11190-11196.
20. Olenych, S. G.; Moussallem, M. D.; Salloum, D. S.; Schlenoff, J. B.; Keller, T. C., Fibronectin and cell attachment to cell and protein resistant polyelectrolyte surfaces. *Biomacromolecules* **2005,** *6* (6), 3252-3258.
21. Lin, C.-A. J.; Sperling, R. A.; Li, J. K.; Yang, T.-Y.; Li, P.-Y.; Zanella, M.; Chang, W. H.; Parak, W. J., Design of an Amphiphilic Polymer for Nanoparticle Coating and Functionalization. *Small* **2008,** *4* (3), 334-341.
22. Carrillo-Carrion, C.; Parak, W. J., Design of pyridyl-modified amphiphilic polymeric ligands: Towards better passivation of water-soluble colloidal quantum dots for improved optical performance. *J. Colloid Interface Sci.* **2016,** *478*, 88 96.
23. Pellegrino, T.; Manna, L.; Kudera, S.; Liedl, T.; Koktysh, D.; Rogach, A. L.; Keller, S.; Rädler, J.; Natile, G.; Parak, W. J., Hydrophobic Nanocrystals Coated with an Amphiphilic Polymer Shell: A General Route to Water Soluble Nanocrystals. *Nano Lett.* **2004,** *4* (4), 703-707.
24. Harder, P.; Grunze, M.; Dahint, R.; Whitesides, G. M.; Laibinis, P. E., Molecular Conformation in Oligo(ethylene glycol)-Terminated Self-Assembled Monolayers on Gold and Silver Surfaces Determines Their Ability To Resist Protein Adsorption. *J. Phys. Chem. B.* **1998,** *102* (2), 426-436.
25. Walkey, C. D.; Olsen, J. B.; Guo, H.; Emili, A.; Chan, W. C., Nanoparticle size and surface chemistry determine serum protein adsorption and macrophage uptake. *J Am Chem Soc* **2012,** *134* (4), 2139-47.
26. Natte, K.; Friedrich, J. F.; Wohlrab, S.; Lutzki, J.; von Klitzing, R.; Osterle, W.; Orts-Gil, G., Impact of polymer shell on the formation and time evolution of nanoparticle-protein corona. *Colloids Surf. B* **2013,** *104*, 213-220.
27. Chun_Ke, P.; Lin, S.; Parak, W. J.; Davis, T. P.; Caruso, F., A decade of the protein corona. *ACS Nano* **2017,** *11* (12), 11773-11776.
28. del_Pino, P.; Pelaz, B.; Zhang, Q.; Maffre, P.; Nienhaus, G. U.; Parak, W. J., Protein corona formation around nanoparticles-from the past to the future. *Mater. Horizons* **2014,** *1*, 301-313.
29. Cedervall, T.; Lynch, I.; Lindman, S.; Berggård, T.; Thulin, E.; Nilsson, H.; Dawson, K. A.; Linse, S., Understanding the Nanoparticle-Protein Corona Using Methods to Quantify Exchange Rates and Affinities of Proteins for Nanoparticles. *Proc. Natl. Acad. Sci. U. S. A.* **2007,** *104* (7), 2050-2055.
30. Casals, E.; Pfaller, T.; Duschl, A.; Oostingh, G. J.; Puntes, V. F., Time Evolution of the Nanoparticle Protein Corona. *ACS Nano* **2010,** *4* (7), 3623-3632.
31. Mahmoudi, M.; Lynch, I.; Ejtehadi, M. R.; Monopoli, M. P.; Bombelli, F. B.; Laurent, S., Protein-Nanoparticle Interactions: Opportunities and Challenges. *Chem. Rev.* **2011,** *111* (9), 5610-5637.
32. Dobrovolskaia, M.; Patri, A.; Zheng, J.; Clogston, J.; Ayub, N.; Aggarwal, P.; Neun, B.; Hall, J.; McNeil, S., Interaction of colloidal gold nanoparticles with human blood: effects on particle size and analysis of plasma protein binding profiles. *Nanomed.: Nanotechnol. Biol. Med.* **2009,** *5* (2), 106-117.
33. Höeg, F.; Schulz, J.; Graf, S.; Salah, D.; Chandralingam, S.; Maison, W.; Parak, W. J.; Schulz, F., Defined Coadsorption of Prostate Cancer Targeting Ligands and PEG on Gold Nanoparticles for Significantly Reduced Protein Adsorption in Cell Media. *J. Phys. Chem. C* **2022,** *126*, 20594-20604.
34. Karakoti, A. S.; Das, S.; Thevuthasan, S.; Seal, S., PEGylated inorganic nanoparticles. *Angew Chem Int Ed Engl* **2011,** *50* (9), 1980-94.
35. Otsuka, H.; Nagasaki, Y.; Kataoka, K., PEGylated nanoparticles for biological and pharmaceutical applications. *Adv. Drug Deliv. Rev.* **2003,** *55* (3), 403-419.
36. Lee, H.; Larson, R. G., Adsorption of Plasma Proteins onto PEGylated Lipid Bilayers: The Effect of PEG Size and Grafting Density. *Biomacromolecules* **2016,** *17* (5), 1757-1765.

37. Yang, Q.; Jones, S. W.; Parker, C. L.; Zamboni, W. C.; Bear, J. E.; Lai, S. K., Evading Immune Cell Uptake and Clearance Requires PEG Grafting at Densities Substantially Exceeding the Minimum for Brush Conformation. *Mol. Pharm.* **2014,** *11* (4), 1250-1258.
38. Susumu, K.; Oh, E.; Delehanty, J. B.; Blanco-Canosa, J. B.; Johnson, B. J.; Jain, V.; Hervey, W. J.; Algar, W. R.; Boeneman, K.; Dawson, P. E.; Medintz, I. L., Multifunctional Compact Zwitterionic Ligands for Preparing Robust Biocompatible Semiconductor Quantum Dots and Gold Nanoparticles. *J. Am. Chem. Soc.* **2011,** *133* (24), 9480-9496.
39. Schlenoff, J. B., Zwitteration: Coating Surfaces with Zwitterionic Functionality to Reduce Nonspecific Adsorption. *Langmuir* **2014,** *30* (32), 9625–9636.
40. Ashraf, S.; Park, J.; Bichelberger, M.; Kantner, K.; Hartmann, R.; Maffre, P.; Said, A. H.; Feliu, N.; Lee, J.; Lee, D.; Nienhaus, G. U.; Kim, S.; Parak, W. J., Zwitterionic surface coating of quantum dots reduces protein adsorption and cellular uptake. *Nanoscale* **2016,** *10*, 1318-1328.
41. Breus, V. V.; Heyes, C. D.; Tron, K.; Nienhaus, G. U., Zwitterionic Biocompatible Quantum Dots for Wide pH Stability and Weak Nonspecific Binding to Cells. *Acs Nano* **2009,** *3* (9), 2573-2580.
42. Dridi, N.; Jin, Z.; Perng, W.; Mattoussi, H., Probing Protein Corona Formation around Gold Nanoparticles: Effects of Surface Coating. *ACS Nano* **2024**.
43. Ostuni, E.; Chapman, R. G.; Holmlin, R. E.; Takayama, S.; Whitesides, G. M., A Survey of Structure−Property Relationships of Surfaces that Resist the Adsorption of Protein. *Langmuir* **2001,** *17* (18), 5605-5620.
44. Sin, M.-C.; Chen, S.-H.; Chang, Y., Hemocompatibility of zwitterionic interfaces and membranes. *Polym. J.* **2014,** *46* (8), 436-443.
45. Estephan, Z.; Schlenoff, P.; Schlenoff, J., Zwitteration As an Alternative to PEGylation. *LANGMUIR* **2011,** *27* (11), 6794-6800.
46. Estephan, Z. G.; Jaber, J. A.; Schlenoff, J. B., Zwitterion-Stabilized Silica Nanoparticles: Toward Nonstick Nano. *Langmuir* **2010,** *26* (22), 16884-16889.
47. Valdeperez, D.; Wutke, N.; Ackermann, L.-M.; Parak, W. J.; Klapper, M.; Pelaz, B., Colloidal stability of polymer coated zwitterionic Au nanoparticles in biological media. *Inorg. Chim. Acta* **2022,** *534*, 120820.
48. Delille, F.; Balloul, E.; Hajj, B.; Hanafi, M.; Morand, C.; Xu, X. Z.; Dumas, S.; Coulon, A.; Lequeux, N.; Pons, T., Sulfobetaine-Phosphonate Block Copolymer Coated Iron Oxide Nanoparticles for Genomic Locus Targeting and Magnetic Micromanipulation in the Nucleus of Living Cells. *Nano Letters* **2023,** *23* (13), 5919–5926.
49. Toro-Mendoza, J.; Maio, L.; Gallego, M.; Otto, F.; Schulz, F.; Parak, W. J.; Sanchez-Cano, C.; Coluzza, I., Bioinspired Polyethylene Glycol Coatings for Reduced Nanoparticle–Protein Interactions. *ACS Nano* **2023,** *17*, 955-965.
50. Brust, M.; Walker, M.; Bethell, D.; Schiffrin, D. J.; Whyman, R., Synthesis of Thiol-Derivatized Gold Nanoparticles in a 2-Phase Liquid-Liquid System. *J. Chem. Soc., Chem. Commun.* **1994,** *1* (7), 801-802.
51. Bastus, N. G.; Comenge, J.; Puntes, V., Kinetically Controlled Seeded Growth Synthesis of Citrate-Stabilized Gold Nanoparticles of up to 200 nm: Size Focusing versus Ostwald Ripening. *Langmuir* **2011,** *27* (17), 11098-11105.
52. Gorzelle, B. M.; Hoffman, A. K.; Keyes, M. H.; Gray, D. N.; Ray, D. G.; Sanders, C. R., Amphipols Can Support the Activity of a Membrane Enzyme. *J. Am. Chem. Soc.* **2002,** *124* (39), 11594-11595.
53. Ghosh, S. K.; Pal, T., Interparticle Coupling Effect on the Surface Plasmon Resonance of Gold Nanoparticles: From Theory to Applications. *Chem. Rev.* **2007,** *107*, 4797-4862.
54. Sperling, R. A.; Liedl, T.; Duhr, S.; Kudera, S.; Zanella, M.; Lin, C.-A. J.; Chang, W. H.; Braun, D.; Parak, W. J., Size Determination of (Bio-) Conjugated Water-Soluble Colloidal Nanoparticles: A Comparison of Different Techniques. *J. Phys. Chem. C* **2007,** *111* (31), 11552-11559.
55. Priebe, J. P.; Satnami, M. L.; Tondo, D. W.; Souza, B. S.; Priebe, J. M.; Micke, G. A.; Costa, A. C. O.; Fiedler, H. D.; Bunton, C. A.; Nome, F., The Chameleon-like Nature of Zwitterionic Micelles: The Intrinsic Relationship of Anion and Cation Binding in Sulfobetaine Micelles. *J. Phys. Chem. B.* **2008,** *112* (46), 14373-14378.

56. Wu, L.; Jasinski, J.; Krishnan, S., Carboxybetaine, sulfobetaine, and cationic block copolymer coatings: A comparison of the surface properties and antibiofouling behavior. *J. Appl. Polym. Sci.* **2012,** *124* (3), 2154-2170.
57. Guthrie, J. P., Hydrolysis of esters of oxy acids: pKa values for strong acids; Brønsted relationship for attack of water at methyl; free energies of hydrolysis of esters of oxy acids; and a linear relationship between free energy of hydrolysis and pKa holding over a range of 20 pK units. *Can. J. Chem.* **1978,** *56* (17), 2342-2354.
58. del_Pino, P.; Yang, F.; Pelaz, B.; Zhang, Q.; Kantner, K.; Hartmann, R.; Baroja, N. M. d.; Gallego, M.; Möller, M.; Manshian, B. B.; Soenen, S. J.; Riedel, R.; Hampp, N.; Parak, W. J., Basic Physicochemical Properties of Polyethylene Glycol Coated Gold Nanoparticles that Determine Their Interaction with Cells. *Angewandte Chemie International Edition* **2016,** *55*, 5483 - 5487.
59. Rana, S.; Yu, X.; Patra, D.; Moyano, D. F.; Miranda, O. R.; Hussain, I.; Rotello, V. M., Control of Surface Tension at Liquid–Liquid Interfaces Using Nanoparticles and Nanoparticle–Protein Complexes. *Langmuir* **2012,** *28* (4), 2023-2027.
60. Sigal, G. B.; Mrksich, M.; Whitesides, G. M., Effect of Surface Wettability on the Adsorption of Proteins and Detergents. *J. Am. Chem. Soc.* **1998,** *120* (14), 3464-3473.
61. Silin, V.; Weetall, H.; Vanderah, D. J., SPR Studies of the Nonspecific Adsorption Kinetics of Human IgG and BSA on Gold Surfaces Modified by Self-Assembled Monolayers (SAMs). *J. Colloid Interface Sci.* **1997,** *185* (1), 94-103.
62. Hua, X. Y.; Rosen, M. J., Dynamic surface tension of aqueous surfactant solutions: I. Basic paremeters. *Journal of Colloid and Interface Science* **1988,** *124* (2), 652-659.
63. Röcker, C.; Pötzl, M.; Zhang, F.; Parak, W. J.; Nienhaus, G. U., A Quantitative Fluorescence Study of Protein Monolayer Formation on Colloidal Nanoparticles. *Nat. Nanotechnol.* **2009,** *4* (9), 577-580.
64. Liedl, T.; Keller, S.; Simmel, F. C.; Rädler, J. O.; Parak, W. J., Fluorescent Nanocrystals as Colloidal Probes in Complex Fluids Measured by Fluorescence Correlation Spectroscopy. *Small* **2005,** *1* (10), 997-1003.
65. Hill, A. V.; Brown, T. G.; Roaf, H. E., The possible effects of the aggregation of the molecules of hemoglobin on its dissociation curves. *The Journal of Physiology* **1910,** *40* (Suppl), i-vii.
66. Fleischer, C. C.; Payne, C. K., Nanoparticle Surface Charge Mediates the Cellular Receptors Used by Protein–Nanoparticle Complexes. *J. Phys. Chem. B.* **2012,** *116* (30), 8901-8907.
67. Fleischer, C. C.; Payne, C. K., Nanoparticle–Cell Interactions: Molecular Structure of the Protein Corona and Cellular Outcomes. *Acc. Chem. Res.* **2014,** *47* (8), 2651-2659.
68. Hühn, D.; Kantner, K.; Geidel, C.; Brandholt, S.; De Cock, I.; Soenen, S. J. H.; Rivera Gil, P.; Montenegro, J.-M.; Braeckmans, K.; Müllen, K.; Nienhaus , G. U.; Klapper, M.; Parak, W. J., Polymer-Coated Nanoparticles Interacting with Proteins and Cells: Focusing on the Sign of the Net Charge. *ACS Nano* **2013,** *7* (4), 3253-3263.
69. Debayle, M.; Balloul, E.; Dembele, F.; Xu, X.; Hanafi, M.; Ribot, F.; Monzel, C.; Coppey, M.; Fragola, A.; Dahan, M.; Pons, T.; Lequeux, N., Zwitterionic polymer ligands: an ideal surface coating to totally suppress protein-nanoparticle corona formation? *Biomaterials* **2019,** *219*, 119357.
70. O'Brien, J.; Wilson, I.; Orton, T.; Pognan, F. o., Investigation of the Alamar Blue (resazurin) fluorescent dye for the assessment of mammalian cell cytotoxicity. *European Journal of Biochemistry* **2000,** *267* (17), 5421-5426.
71. Sarker, S. D.; Nahar, L.; Kumarasamy, Y., Microtitre Plate-Based Antibacterial Assay Incorporating Resazurin as an Indicator of Cell Growth, and Its Application in the in Vitro Antibacterial Screening of Phytochemicals. *Methods* **2007,** *42*, 321-324.
72. Brunetti, V.; Chibli, H.; Fiammengo, R.; Galeone, A.; Malvindi, M. A.; Vecchio, G.; Cingolani, R.; Nadeau, J. L.; Pompa, P. P., InP/ZnS as a safer alternative to CdSe/ZnS core/shell quantum dots: in vitro and in vivo toxicity assessment. *Nanoscale* **2013,** *5* (1), 307-317.
73. Derfus, A. M.; Chan, W. C. W.; Bhatia, S. N., Probing the Cytotoxicity of Semiconductor Quantum Dots. *Nano Lett.* **2004,** *4* (1), 11-18.

74. Kirchner, C.; T., L.; Kudera, S.; Pellegrino, T.; Muñoz Javier, A.; Gaub, H. E.; Stölzle, S.; Fertig, N.; Parak, W. J., Cytotoxicity of Colloidal CdSe and CdSe/ZnS Nanoparticles. *Nano Lett.* **2005,** *5* (2), 331-338.
75. Alkilany, A. M.; Nagaria, P. K.; Hexel, C. R.; Shaw, T. J.; Murphy, C. J.; Wyatt, M. D., Cellular uptake and cytotoxicity of gold nanorods: molecular origin of cytotoxicity and surface effects. *Small* **2009,** *5* (6), 701-8.
76. Alkilany, A.; Murphy, C., Toxicity and cellular uptake of gold nanoparticles: what we have learned so far? *Journal of Nanoparticle Research* **2010,** *12* (7), 2313-2333.
77. Bhattacharjee, S.; de Haan, L. H.; Evers, N. M.; Jiang, X.; Marcelis, A. T.; Zuilhof, H.; Rietjens, I. M.; Alink, G. M., Role of surface charge and oxidative stress in cytotoxicity of organic monolayer-coated silicon nanoparticles towards macrophage NR8383 cells. *Part Fibre Toxicol* **2010,** *7*, 25.
78. Frohlich, E., The role of surface charge in cellular uptake and cytotoxicity of medical nanoparticles. *International Journal of Nanomedicine* **2012,** *7*, 5577-5591.
79. Breus, V. V.; Pietuch, A.; Tarantola, M.; Basche, T.; Janshoff, A., The effect of surface charge on nonspecific uptake and cytotoxicity of CdSe/ZnS core/shell quantum dots. *Beilstein Journal of Nanotechnology* **2015,** *6*, 281-292.
80. Allouni, Z. E.; Gjerdet, N. R.; Cimpan, M. R.; Hol, P. J., The effect of blood protein adsorption on cellular uptake of anatase TiO2 nanoparticles. *International Journal Of Nanomedicine* **2015,** *10*, 687-695.
81. Johnston, H. J.; Semmler-Behnke, M.; Brown, D. M.; Kreyling, W.; Tran, L.; Stone, V., Evaluating the uptake and intracellular fate of polystyrene nanoparticles by primary and hepatocyte cell lines in vitro. *Toxicology And Applied Pharmacology* **2010,** *242* (1), 66-78.
82. Geidel, C.; Schmachtel, S.; Riedinger, A.; Pfeiffer, C.; Müllen, K.; Klapper, M.; Parak, W. J., A General Synthetic Approach for Obtaining Cationic and Anionic Inorganic Nanoparticles *via* Encapsulation in Amphiphilic Copolymers. *Small* **2011,** *7* (20), 2929–2934.
83. Khrenov, V.; Schwager, F.; Klapper, M., The formation of hydrophobic inorganic nanoparticles in the presence of amphiphilic copolymers. *Colloid Polym Sci* **2006,** *28*, 927–934.
84. Fu, K. W., X.; Yuan, X.; Wang, D.; Mi, X.; Tan, X.; Zhang, Y., Size-Dependent Penetration of Gold Nanoprobes into Fixed Cells. *ACS Omega* **2021,** *6* (5), 3791-3799.
85. Tomaszewska, E. R.-S., K.; Sztandera, K.; Błażałek, P.; Głowacki, R.; Janaszewska, A.; Janasik, B.; Celichowski, G.; Wąsowicz, W.; Klajnert-Maculewicz, B.; Grobelny, J., Systematic Studies of Gold Nanoparticles Functionalised with Thioglucose and its Cytotoxic Effect. *ChemistrySelect* **2021,** *6* (6), 1230-1237.
86. Anderson, R. E.; Chan, W. C. W., Systematic Investigation of Preparing Biocompatible, Single, and Small ZnS-Capped CdSe Quantum Dots with Amphiphilic Polymers. *ACS Nano* **2008,** *2* (7), 1341-1352.
87. Pellegrino, T.; Kudera, S.; Liedl, T.; Javier, A. M.; Manna, L.; Parak, W. J., On the Development of Colloidal Nanoparticles towards Multifunctional Structures and their Possible Use for Biological Applications. *Small* **2005,** *1* (1), 48-63.
88. Instruments, D. Pendant drop method — Optical determination of the surface/interfacial tension. https://www.dataphysics-instruments.com/knowledge/understanding-interfaces/pendant-drop-method/# (accessed 13-3-2024).
89. Jasper, J. J., The Surface Tension of Pure Liquid Compounds. *Journal of Physical and Chemical Reference Data* **2009,** *1* (4), 841-1010.
90. Du, Y. J., J.; Liang, H.; Jiang, W., Structural and Physicochemical Properties and Biocompatibility of Linear and Looped Polymer-Capped Gold Nanoparticles. *Langmuir* **2019,** *35* (25), 8316-8324.
91. Maffre, P.; Brandholt, S.; Nienhaus, K.; Shang, L.; Parak, W. J.; Nienhaus, G. U., Effects of surface functionalization on the adsorption of human serum albumin onto nanoparticles - a fluorescence correlation spectroscopy study. *Beilstein J. Nanotechnol.* **2014,** *5*, 2036 2047.
92. Maffre, P.; Nienhaus, K.; Amin, F.; Parak, W. J.; Nienhaus, G. U., Characterization of Protein Adsorption onto FePt Nanoparticles Using Dual-Focus Fluorescence Correlation Spectroscopy. *Beilstein J. Nanotechnol.* **2011,** *2*, 374–383.

93. Shang, L.; Nienhaus, G. U., In Situ Characterization of Protein Adsorption onto Nanoparticles by Fluorescence Correlation Spectroscopy. *Acc. Chem. Res.* **2017,** *50* (2), 387-395.
94. de Thomaz, A. A. A., D. B.; Pelegati, V. B.; Carvalho, H. F.; Cesar, C. L., Measurement of the Hydrodynamic Radius of Quantum Dots by Fluorescence Correlation Spectroscopy Excluding Blinking. *J. Phys. Chem. B.* **2015,** *119* (11), 4294-4299.
95. Chakraborty, I.; Feliu, N.; Roy, S.; Dawson, K.; Parak, W. J., Protein-Mediated Shape-Control of Silver Nanoparticles. *Bioconjugate Chem.* **2018,** *29*, 1261–1265.
96. Ma, X.; Hartmann, R.; Aberasturi, D. J. d.; Yang, F.; Soenen, S. J. H.; Manshian, B. B.; Franz, J.; Valdeperez, D.; Pelaz, B.; Feliu, N.; Hampp, N.; Riethmüller, C.; Vieker, H.; Frese, N.; Gölzhäuser, A.; Simonich, M.; Tanguay, R. L.; Liang, X.-J.; Parak, W. J., Colloidal Gold Nanoparticles Induce Changes in Cellular and Subcellular Morphology. *ACS Nano* **2017,** *11*, 7807−7820.
